# Metatranscriptomic assessment of diarrhoeic faeces reveals diverse RNA viruses in rotavirus group A infected piglets and calves from India

**DOI:** 10.3389/fcimb.2023.1258660

**Published:** 2023-10-27

**Authors:** Pradeep Sawant, Abhijeet Kulkarni, Rajkumar Mane, Renu Patil, Mallika Lavania

**Affiliations:** ^1^ Enteric Viruses Group, Indian Council of Medical Research (ICMR) - National Institute of Virology, Pune, India; ^2^ Bioinformatics Centre, Savitribai Phule Pune University, Pune, Maharashtra, India

**Keywords:** RNA virome, diarrhoea, rotavirus group A, astrovirus, picornavirus

## Abstract

RNA viruses are a major group contributing to emerging infectious diseases and neonatal diarrhoea, causing morbidity and mortality in humans and animals. Hence, the present study investigated the metatranscriptomic-derived faecal RNA virome in rotavirus group A (RVA)-infected diarrheic piglets and calves from India. The viral genomes retrieved belonged to *Astroviridae* in both species, while *Reoviridae and Picornaviridae* were found only in piglets. The nearly complete genomes of porcine RVA (2), astrovirus (AstV) (6), enterovirus G (EVG) (2), porcine sapelovirus (PSV) (2), Aichivirus C (1), and porcine teschovirus (PTV) (1) were identified and characterised. In the piglet, AstVs of PAstV2 (MAstV-26) and PAstV4 (MAstV-31) lineages were predominant, followed by porcine RVA, EVG, PSV, Aichivirus C, teschovirus (PTV-17) in decreasing order of sequence reads. In contrast, AstV accounted for the majority of reads in bovines and belonged to MAstV-28 and a proposed MAstV-35. Both RVA G4P[6] strains exhibited prototype Gottfried strains like a genotypic constellation of G4-P[6]-I1-R1-C1-M1-A8-N1-T1-E1-H1. Ten out of eleven genes were of porcine origin, while the VP7 gene clustered with G4-lineage-1, consisting of human strains, suggesting a natural porcine-human reassortant. In the recombination analysis, multiple recombination events were detected in the PAstV4 and PAstV2 genomes, pointing out that these viruses were potential recombinants. Finally, the study finds diverse RNA virome in Indian piglets and calves for the first time, which may have contributed to diarrhoea. In the future, the investigation of RNA virome in animals will help in revealing pathogen diversity in multifactorial diseases, disease outbreaks, monitoring circulating viruses, viral discovery, and evaluation of their zoonotic potential.

## Highlights

* The exploration of faecal RNA virome in rotavirus group A (RVA)-positive piglets and calves revealed viruses from the *Reoviridae, Astroviridae*, and *Picornaviridae* families.

* The predominant virus in piglets were rotavirus and astrovirus while only astrovirus in calves.

* The whole genome of the G4P[6] strain resembling prototype Gottfried genotype constellation G4-P[6]-I1-R1-C1-M1-A8-N1-T1-E1-H1 was found to be porcine-human reassortant.

* First full genome sequences of porcine AstV strains (lineage 4 and lineage 2), the nearly complete genome of porcine teschovirus, and partial genomes of bovine AstV

* The potential recombination events were detected in PAstV2 (MAstV-26) and PAstV4 (MAstV-31)

## 1 Introduction

RNA viruses, the most important group involved in zoonotic infections, pose a challenge for safeguarding public and animal health. RNA viruses, because of their broad host range, great genetic diversity, higher mutation rate, and recombination capacity, often emerge as new viruses (Papp et al., 2013a; Shi et al., 2018; Werid et al., 2022). The major RNA viruses causing diarrhoea including rotavirus A-C and H, porcine epidemic diarrhoea virus, and transmissible gastroenteritis virus, are in pigs, while rotavirus A-C, bovine viral diarrhoea virus, and coronavirus are in calves. Among them, rotavirus is a major enteric pathogen causing diarrhoea (Crawford et al., 2017). Other RNA viruses, such as caliciviruses (norovirus, sapovirus), astroviruses (AstVs), picornaviruses (kobuvirus, teschovirus, enteroviruses), and toroviruses, are detected as coinfections or superinfections with or without clinical symptoms (Su et al., 2020). Some of these porcine and bovine viruses are genetically more similar to human viruses and are considered potentially zoonotic (Papp et al., 2013b; Werid et al., 2022).

The RVA is a non-enveloped virion with a triple-layered capsid surrounding 11 double-stranded RNA genome segments, which encode six structural (VP1-VP4, VP6, and VP7) and five or six non-structural (NSP1-NSP5/6) proteins. The traditional RVA classification using nucleotide changes in VP7 (Genotypes) and VP4 (P genotypes) gene segments has assigned 41 G and 57 P types in humans and animals (https://rega.kuleuven.be/cev/viralmetagenomics/virus-classification). To understand the genetic relatedness between human and animal RVA, a whole genome classification system assigning a specific genotype to each of the 11 gene segments was adopted. Accordingly, human RVAs belong to three genogroups: Wa, DS-1, and AU-1 (Matthijnssens et al., 2008a; Matthijnssens et al., 2008b). The Wa-like genogroup bears the constellations Gx-P[x]-I1-R1-C1-M1-A1-N1-T1-E1-H1, DS-1-like of Gx-P[x]-I2-R2-C2-M2-A2-N2-T2-E2-H2, and AU-1-like of I3-R3-C3-M3-A3-N3-T3-E3-H3 (Matthijnssens et al., 2008a; Matthijnssens et al., 2008b). In pigs, the G4P[6] combination with the genotype constellation G4-P[6]-I1/I5-R1-C1-M1-A8-N1-T1-E1-H1/H2 is detected throughout the world (Malasao et al., 2018). These genotype constellations are also detected in humans as either porcine-human reassortant or direct transmission of the porcine virus (Tacharoenmuang et al., 2021).

AstVs are small, non-enveloped viruses consisting of single-stranded, positive-sense RNA genomes that contain three open reading frames (ORFs) designated as ORF1a coding for nonstructural proteins, ORF1b for RNA dependent RNA polymerase (RdRp), and ORF2 for capsid. The family *Astroviridae* is divided into the genera Mamastrovirus (MAstV), which infects mammals, and Avastrovirus, which infects birds (Shan et al., 2011). According to the latest International Committee on Taxonomy of Viruses, 2012, MAstV includes 19 established genotypes (MAstV-1 to 19), 14 proposed genotypes (MAstV-20 to 33), and undefined genotypes. Porcine astrovirus (PAstV) lineages 1-5 correspond to seven genotype species: MAstV-3 (PAstV1), MAstV-22 (PAstV3), MAstV-24 (PAstV5), MAstV-26 and -27 (PAstV4), MAstV-31 and -32 (PAstV2), and bovine astrovirus (BAstV) belong to five genotype species (MAstV-13, -28 to 30, and -33) (Boujon et al., 2017; Zhu et al., 2021). AstVs have a wide geographic distribution and are commonly detected in healthy and diarrheic animals. These viruses are increasingly detected as neuroinvasive pathogens in various animal and avian species, including humans (Giannitti et al., 2019). Despite the lack of evidence of zoonosis, their genetic similarity with human AstV, recombination potential, and concurrent detection in sewage make them emerging potentially zoonotic viruses (Ibrahim et al., 2022; Werid et al., 2022).

‘Porcine enteroviruses’ members of the family *Picornavirdae* constitute viruses from genera porcine teschovirus (PTV1-13), enterovirus (EVG1-20), sapelovirus (SV-A) and kobuvirus (Aichivirus C). The PTV infections in endemic situations are asymptomatic; however, some virulent strains cause neurologic disease, mild (Talfan disease) or severe (Teschen disease), and a range of systemic infections, including diarrhoea, respiratory illness, and myocarditis. Porcine sapeloviruses (PSV) are causative agents of diarrhoea, respiratory and reproductive presentations, and more recently, polioencephalomyelitis. The fourth virus, Aichivirus C (Genus Kobuvirus), is detected in healthy and diarrheic piglets, and it is suggested that it can induce diarrhoea in the presence of viruses like rotavirus and transmissible gastroenteritis (Cao et al., 2012). The zoonotic potential is suggested for kobuviruses based on their homology with human kobuviruses and the transmission of bovine kobuvirus to pigs (Ibrahim et al., 2022; Werid et al., 2022).

The global knowledge of RNA virus diversity is limited by dependence on cell culture and reverse transcriptase polymerase chain reaction (RT-PCR) for virus discovery, priority on virus pathogens in humans, or economic importance in animals (Chen et al., 2022). Fortunately, the metatranscriptome, which detects transcribed RNA, has proved to be a powerful tool for discovering the entire virome of humans, animals, and plants. At present, only a few studies have explored the enteric RNA virome of piglets and calves and none from India (Chen et al., 2018; Cortey et al., 2019; Smoľak et al., 2022). However, a wide knowledge gap exists on the diversity of animal RNA viruses in India. Since RNA viruses have the potential to inflict severe disease outbreaks and have zoonotic importance, unraveling their diversity is of immense importance for the safeguarding of human and animal health. The present study employed the metatranscriptomic approach to detect and recover the whole genomes of some previously uncharacterised viruses in diarrheic Indian piglets and calves.

## 2 Materials and methods

### 2.1 Sample preparation and nucleic acid extraction

The faecal samples were randomly collected for the period 2017 to 2019 from diarrheic and apparently healthy piglets and calves under six months of age from Chandkhed (22) and Tathawade (17) in Western Maharashtra, India. All the samples were transported on ice packs and stored at -70°C. The samples were tested for RVA by VP6 based RT-PCR using previously published primers (Iturriza Gómara et al., 2002). The representative RVA positive samples from piglets (NIV1740786 and NIV1740787) and calves (NIV198014 & NIV198016) were then enriched for viral particles by the NetoVIR (Novel Enrichment Technique of VIRomes) technique (Conceiço-Neto et al., 2018). The faecal suspensions (10%) were made simply by vortexing 50 mg of the faeces with 500 µl of sterile phosphate buffer saline in a clean 1.5 ml microcentrifuge tube. After that, they were centrifuged for 3 minutes at 17000 g. A 0.22µm filter (Merck Millipore, Burlington, MA, United States) was used to filter the supernatant. After that, for removal of the free nucleic acids, the filtrate was incubated for 2 hours at 37°C with a combination of benzonase (Novagen, Madison, WI, United States), micrococcal nuclease (New England Biolabs, MA, United States), and 7 µl of homemade buffer (20 mM Tris, 100 mM CaCl_2_, and 30 mM MgCl_2_, pH = 8). By adding 7µl of 10 nM EDTA, the process was stopped. The QIAAmp^®^ Viral RNA Mini kit (Qiagen, Hilden, Germany) was used to extract the nucleic acids as directed by the manufacturer without the use of carrier RNA.

### 2.2 Library preparation and sequencing

At Genotypic Technology Pvt. Ltd., Bangalore, India, RNA sequencing libraries were prepared using the NEBNext^®^ UltraTM II Directional RNA Library Prep Kit (New England BioLabs, MA, USA), which is compatible with the Illumina platform. For priming and fragmentation, 50ng of total RNA was collected. Further, first-strand cDNA synthesis and second-strand cDNA synthesis were applied to fragmented and primed RNA. Using JetSeq Beads (Bioline, Cat. No. BIO-68031), the double-stranded cDNA was purified. Following end-repair, adenylation, and ligation of purified cDNA to Illumina multiplex barcode adapters by the NEBNext^®^ UltraTM II Directional RNA Library Prep methodology, second-strand excision was performed using the USER enzyme at 37°C for 15 minutes. Illumina Universal Adapters (5’-AATGATACGGCGACCGAGATCTACACTTTCCCTACACGACGCTCTTCCGATCT-3’) and Index Adapter (5’-GATCGGAAGAGCACACGTCTGAACTCCAGTCAC [INDEX] ATCTCGTATGCCGTTCTGCTTG-3’) have been used in the study. JetSeq Beads were used to purify the adapter-ligated cDNA, and 11 cycles of indexing (98°C for 30 sec) and cycling (98°C for 10 sec, 65°C for 75 sec, and 65°C for 5 min) were used to enrich the adapter-ligated fragments. Following a library-quality control check, JetSeq Beads were used to purify the final PCR result (sequencing libraries). The Qubit fluorometer (Thermo Fisher Scientific, MA, USA) was used to quantify Illumina-compatible sequencing libraries, and the Agilent 2200 Tapestation was used to examine the fragment size distribution. Using the Illumina Hiseq (150 x 2 chemistry) instrument, libraries were sequenced (**Supplementary File 1**).

### 2.3 Sequence analysis

Sequencing was performed on the Illumina platform in pair-end sequencing format for all four samples. The raw data files were subjected to quality checks using the Linux-compatible FastQC v0.11.9 tool. The low-quality reads, adapters, and primers were removed with the Trimmomatic tool available on the Galaxy server (https://usegalaxy.org/). The cleaned reads were classified with the Kraken viral 2020 database using the Kraken tool (version 1.3.1) with default parameters on the Galaxy server. The Krona tools (version 2.7.1) on Galaxy server were then used to generate the interactive charts for the hierarchical classification and visualization of classified viruses in pie charts. For the further characterization of the virus, all de-multiplexed reads of an interested taxon were filtered, extracted, and *de novo* assembled with the Shovill Faster SPAdes assembler on the Galaxy server (version 1.1.0+galaxy1) with the assembly options for paired-end reads. For further validation and identification, the assembled contigs from each viral sample were annotated using the standalone blast (NCBI-BLAST+” tool v2.9.0) with a custom viral genome sequence database.

### 2.4 Phylogenetic analysis

The VP7 and VP4 (RVA), ORF1a, ORF1b, and ORF2 (AstV), and polyprotein (EVG, PSV, Aichivirus C, and PTV) nucleotide/amino acid sequences of detected viruses were aligned with the same gene or region of prototype strains with the help of the online MAFTT v7 program with default parameters. The phylogenetic trees were constructed by the neighbour-joining tree using the Kimura 2-parameter model (nucleotide sequences) and the P Distance Model (amino acid sequences) in MEGA 11.0 software (Tamura et al., 2021). The reliability of different phylogenetic groupings was confirmed by 1000 bootstrap replications, and amino acid distances were calculated using the P Distance Model.

### 2.5 Recombination analysis

The nucleotide sequences of the complete genomes of the detected viruses were aligned with datasets used in phylogenetic analysis to detect recombinants. Then SIMPLOT v3.5.1. was used to identify potential recombination sites (Lole et al., 1999). The bootscaning in the same programme was applied to identify the phylogenetically informative sites supporting alternative tree topologies (Salminen et al., 1995).

## 3 Results

### 3.1 Mixed RNA viruses were found in RVA-positive samples

**Figure 1:**
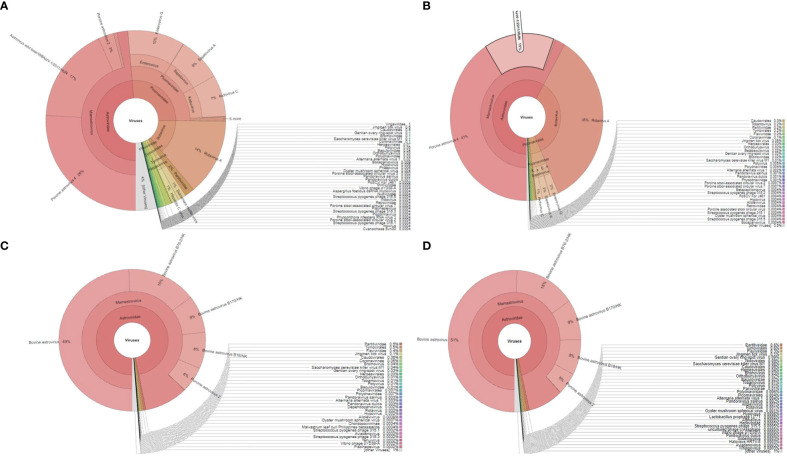
The Krona plot shows taxonomically classified detected reads from each faecal sample and viruses reported as pie charts: **(A, B)**, porcine sample number NIV1740786 and NIV1740787; **(C, D)**, bovine sample number NIV198014 and NIV198016.

We were able to generate the raw Illumina reads for four samples in the range of 2.2-4.9 million. The final processed reads, after adapter trimming and mapping against their respective host genomes, for NIV1740786, NIV1740787, NIV198014, and NIV198016 samples were 235506, 270336, 460987, and 371965, respectively. The reads from each sample were taxonomically classified and visualized as pie charts by using Kraken and Krona tools. In the porcine samples, NIV1740786 & NIV1740787, reads of PAstV, porcine RVA, porcine enterovirus G (data unpublished), PSV, Aichivirus C, and PTV, made up, of 48.59%, 14%, 10%, 8%, 7%, 0.8%, and 58.27%, 35%, 2%, 2%, 1%, and 0.08%, respectively, of all aligned reads (**Figures 1A, B**). In the bovine samples, NIV198014 and NIV198016, sequences belonging to BAstV and bovine RVA, made up 97.02%, 0.002%, and 96.38%, 0.001% of all aligned reads (**Figures 1C, D**). Upon BLASTn analysis, the final next-generation sequencing (NGS) reads showed homologies to several porcine and bovine viruses (**Table 1**; **Supplementary File 2**). A large number of reads were labeled as uncategorised. Further analysis of these reads may reveal previously unidentified viruses. The sequence data generated in the present study were deposited in the NCBI Sequence Read Archive, https://www.ncbi.nlm.nih.gov/sra (Accession number: PRJNA941130).

**Table 1:** The diversity of sequences identified, their per cent identity, and coverage in porcine and bovine faecal samples.

Sample Number	Virus detected	qaccver	saccver	pident	length	mismatch	gapopen	qstart	qend	sstart	send	bitscore	evalue	qcovs
NIV1740786 (Porcine)	*Kobuvirus*	contig00001	KM977675.1	89.713	8049	809	18	4	8043	8076	38	10259	0	99
	*Sapelovirus*	contig00002	KJ821021.1	87.778	7503	910	7	1	7499	25	7524	8769	0.00E+00	100
	*Teschovirus*	contig00003	MW145169.1	86.295	6939	934	17	107	7038	1	6929	7529	0.00E+00	98
	*Astrovirus*	contig00004	KX060809.1	84.711	6678	972	39	2	6656	6652	1	6630	0.00E+00	99
		contig00005	OM104029.1	89.013	4396	468	12	2250	6636	4390	1	5428	0.00E+00	66
		contig00006	MW784083.1	83.34	5204	830	30	7	5190	6	5192	4772	0.00E+00	86
	*Enterovirus G*	contig00007	MN734577.1	99.787	3286	2	3	1	3281	3303	18	6024	0.00E+00	99
	*Rotavirus A (VP2)*	contig00008	GU199192.1	97.157	2638	73	2	56	2692	2647	11	4455	0.00E+00	98
	*Rotavirus A (VP3)*	contig00009	MT876640.1	96.405	2587	93	0	1	2587	4	2590	4263	0.00E+00	100
	*Picobirnavirus*	contig00010	MW977860.1	79.087	2453	443	56	7	2422	2427	8	1624	0.00E+00	99
	*Rotavirus A (VP1)*	contig00011	KM393170.1	97.285	1989	54	0	1	1989	3284	1296	3374	0.00E+00	100
	*Rotavirus A (NSP1)*	contig00012	KM393173.1	95.508	1536	69	0	1	1536	1	1536	2455	0.00E+00	100
	*Enterovirus G*	contig00013	MN734577.1	99.497	1392	0	7	1	1390	7378	5992	2525	0.00E+00	100
	*Rotavirus A (VP1)*	contig00014	KM393170.1	97.424	1320	34	0	1	1320	1320	1	2250	0.00E+00	99
	*Rotavirus A (VP4)*	contig00015	KX363402.1	87.622	1131	140	0	7	1137	2352	1222	1314	0.00E+00	99
	*Rotavirus A (VP4)*	contig00016	MN066814.1	95.558	1103	49	0	1	1103	13	1115	1766	0.00E+00	99
	*Enterovirus G*	contig00017	MN734577.1	98.639	1102	13	2	1	1101	4293	3193	1951	0.00E+00	100
	*Rotavirus A (NSP3)*	contig00018	KF726074.1	94.934	1066	54	0	1	1066	10	1075	1670	0.00E+00	100
	*Rotavirus A (VP7)*	contig00019	M86490.1	97.019	1040	31	0	8	1047	1051	12	1749	0.00E+00	99
	*Rotavirus A (NSP2)*	contig00020	KM393174.1	97.209	1039	29	0	1	1039	1053	15	1759	0.00E+00	100
	*Rotavirus A (VP6)*	contig00021	KX638599.1	97.443	1017	26	0	1	1017	1025	9	1735	0.00E+00	100
	*Enterovirus G*	contig00022	MN734577.1	100	954	0	0	1	954	6102	5149	1762	0.00E+00	100
	*Enterovirus G*	contig00023	MN734577.1	99.648	853	0	1	1	850	5147	4295	1555	0.00E+00	100
	*Rotavirus A (NSP4*)	contig00024	AY601542.1	97.771	673	15	0	1	673	732	60	1160	0.00E+00	100
NIV1740787 (Porcine)	*Enterovirus G*	contig00001	MF782664.1	91.48	7570	633	10	1	7567	460	8020	10395	0	100
	*Sapelovirus*	contig00002	KJ821021.1	87.724	7462	909	7	3	7460	44	7502	8698	0.00E+00	99
	*Enterovirus G*	contig00003	MN734577.1	99.363	7375	30	13	4	7367	16	7384	13343	0.00E+00	99
	*Astrovirus*	contig00004	KX060809.1	84.711	6678	972	39	27	6681	6652	1	6630	0.00E+00	99
	*Astrovirus*	contig00005	MW784083.1	83.337	5203	830	30	1221	6403	5192	7	4771	0.00E+00	86
	*Kobuvirus*	contig00006	KT892971.1	87.992	4747	559	7	1	4741	5175	434	5598	0.00E+00	100
	*Astrovirus*	contig00007	OM104029.1	89.694	4124	410	12	6	4120	4118	1	5247	0.00E+00	99
	*Rotavirus A (VP1)*	contig00008	KM393170.1	97.411	3283	85	0	16	3298	1	3283	5592	0.00E+00	99
	*Rotavirus A (VP2)*	contig00009	GU199192.1	97.157	2638	73	2	7	2643	11	2647	4455	0.00E+00	98
	*Astrovirus*	contig00010	OP643775.1	86.06	2561	325	20	52	2601	1	2540	2723	0.00E+00	97
	*Rotavirus A (VP3)*	contig00011	MT876640.1	96.405	2587	93	0	1	2587	2588	2	4263	0.00E+00	100
	*Rotavirus A (VP4)*	contig00012	JX156399.2	87.591	2345	291	0	8	2352	2355	11	2719	0.00E+00	99
	*Rotavirus A (NSP1)*	contig00013	KM393173.1	95.587	1541	68	0	2	1542	1	1541	2470	0.00E+00	99
	*Rotavirus A (VP6)*	contig00014	KX638599.1	97.615	1342	32	0	1	1342	10	1351	2302	0.00E+00	100
	*Rotavirus A (NSP3)*	contig00015	KF726074.1	94.915	1062	54	0	7	1068	1067	6	1663	0.00E+00	96
	*Rotavirus A (VP7)*	contig00016	M86490.1	96.771	1053	33	1	2	1053	1061	9	1755	0.00E+00	99
	*Rotavirus A (NSP2)*	contig00017	KM393174.1	97.19	1032	29	0	1	1032	1056	25	1746	0.00E+00	100
	*Rotavirus A (NSP4)*	contig00018	AY601542.1	97.925	723	15	0	1	723	12	734	1253	0.00E+00	99
	*Rotavirus A (NSP5)*	contig00019	AB741659.1	99.046	629	6	0	1	629	641	13	1129	0.00E+00	100
NIV198014 (Bovine)	*Astrovirus*	contig00002	ON682281.1	76.769	4933	1082	58	38	4940	105	5003	2702	0	98
		contig00003	ON682297.1	86.074	1429	199	0	2	1430	1429	1	1537	0	99
		contig00005	MW373713.1	83.992	1037	166	0	1	1037	2356	1320	996	0	100
		contig00006	ON624268.1	88.714	381	38	5	6	383	6308	5930	460	3.84E-12	37
		contig00011	HQ916314.1	80.891	539	92	10	1	535	3925	4456	414	1.67E-11	93
		contig00012	MK378521.1	84.874	119	18	0	93	211	4527	4645	121	3.83E-23	22
NIV198016 (Bovine)	*Astrovirus*	contig00001	ON624260.1	80.556	2808	527	16	2	2800	1103	3900	2143	0	96
		contig00002	ON624261.1	88.492	252	29	0	38	289	6048	5797	305	2.94E-7	16

### 3.2 Rotavirus A

**Figure 2:**
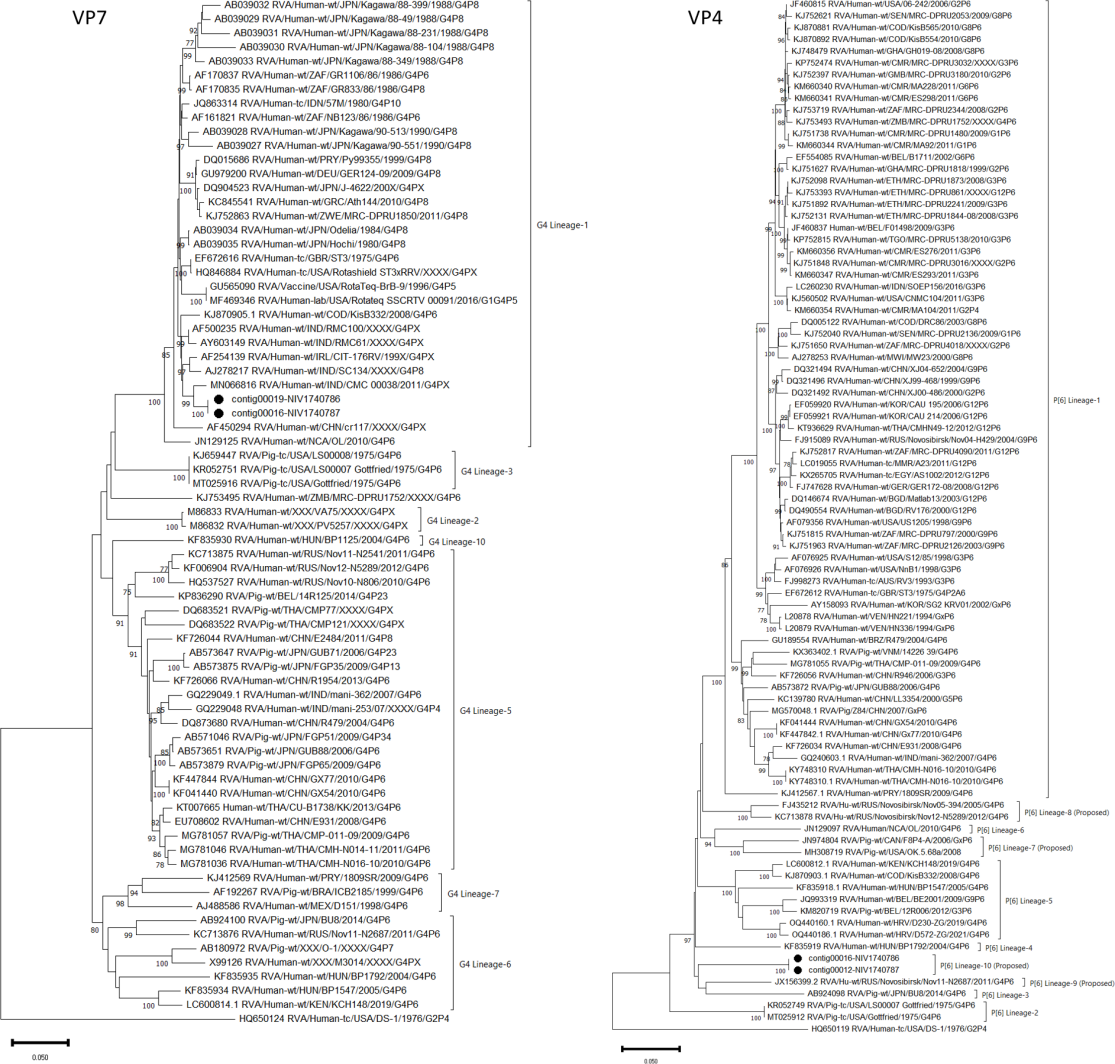
The phylogenetic tree based on nucleotide sequences of the VP7 and VP4 genes of Indian porcine G4P[6] RVA strain: The neighbour-joining tree was established using the Kimura 2-parameter model with 1000 bootstrap replicates in MEGA 11.0 software. The bar represents the genetic distance, while numbers indicate the bootstrap replicates (>75% are shown at branch points). The G4 and P[6] lineages assigned as per previous reports (Wandera et al., 2021). The detected strains are marked with a black circle.

The 11 contigs covering complete or nearly complete sequences of VP7 (contig00019-NIV1740786 & contig00016-NIV1740787), VP4 (contig00012-NIV1740787), VP6 (contig00021-NIV1740786 & contig00014-NIV1740787), VP1 (contig00008-NIV1740787), VP2 (contig00008-NIV1740786 & contig00009-NIV1740787), VP3 (contig00009-NIV1740786 & contig00011-NIV1740787), NSP1 (contig00012-NIV1740786 & contig00013-NIV1740787), NSP2 (contig00020-NIV1740786 & contig00017-NIV1740787), NSP3 (contig00018-NIV1740786 & contig00015-NIV1740787), NSP4 (contig00024-NIV1740786 & contig00018-NIV1740787), NSP5/6 (contig00019-NIV1740787) genes of RVA were retrieved from porcine samples. From the NIV1740786 sample, partial sequences of VP4 (contig00015 & contig00016) and VP1 (contig00011 & contig00014) were retrieved, but the NSP4/5 gene was not recovered (**Table 1**). Both the detected RVA strains were identical in all genes. In contrast, although the reads of the RVA genome were detected in both bovine samples, no contigs were assembled. The per cent amino acid (AA) identities of all the genes with prototype strains, best BLAST hit strains, representative porcine, porcine-like human, and human strains are presented in **Table 2**. Both the porcine NIV strains displayed a prototype Gottfried genotype constellation as G4-P[6]-I1-R1-C1-M1-A8-N1-T1-E1-H1. The VP7 gene shared the highest AA identity with the Indian human strain CMC00038 (G4P[x]) and the prototype human ST3 strain (G4P2A[6]). Phylogenetically, based on nucleotide identity, the G4 genotype is divided into 10 lineages (Wandera et al., 2021). The VP7 gene of the study strain clustered with G4-lineage-1, consisting of human RVA strains from worldwide (**Figure 2**). The VP4 gene belonged to the P[6] genotype and was most similar to the human Russian strain Nov11-N2687 and distantly to the prototype porcine strain Gottfried (G4P[6]). The P[6] genotype consists of six lineages based on nucleotide identity (Wandera et al., 2021). However, after the alignment of VP4 nucleotide sequences of the genotype, the number can be increased to 10 lineages (within-group distance: 91.60%; between-group distance: 93.2%). Accordingly, the VP4 gene of the present study belonged to the proposed P[6] lineage-10 (**Figure 2**). The VP6 gene at the amino acid level was identical to the human strain from Russia (Nov11-N2687). However, at the nucleotide level, the VP6 gene shared the highest identity (94.90- 97.57%) with Indian human (RV1020, CMC_00038, NIV929893) strains and (96.15-96.73%) with Indian porcine (UP-Por30, UP-Por34) strains, which are excluded from full genome analysis because sequence data for a few genes is available in GenBank. The other structural protein-encoding genes, VP1 showed close AA identity with porcine strain RU172 (G12P[P7], while VP2 and VP3 were highly similar with porcine-like human strains, E931 (G4P[6]) and GX77 (G4P[6]), respectively. The nonstructural protein coding genes NSP1 and NSP2 with porcine strain HP113 (G6P[13]), NSP3 with porcine strain BU8 (G4P[6]), NSP4 and NSP5/6 with porcine-like human strains NIV929893 (G1P[19]) and CMH-N016-10 (G4P[6]), respectively, exhibited maximum AA identity.

**Table 2:** The heatmap showing comparative amino acid identities (%) of Indian porcine RVA strains NIV1740786 & NIV1740787 (G4P[6]) with strains detected in porcine and human.

RVA	G4-P[6]-I1-R1-C1-M1-A8-N1-T1-E1-H1										
	VP7	VP4*	VP6	VP1*	VP2	VP3	NSP1	NSP2	NSP3	NSP4	NSP5/6^X^
RVA/Human-tc/USA/Wa/1974/G1P1A8	76.15	75.02	98.99	97.61	97.42	94.37	79.79	90.57	87.79	88.63	93.77
RVA/Human-tc/USA/DS-1/1976/G2P4	71.08	76.09	92.7	89.8	92.25	81.41	69.07	82.49	77.07	81.57	84.68
RVA/Human-tc/GBR/ST3/1975/G4P2A6	98.15	90.49	98.49	96.97	97.53	92.57	82.27	89.73	87.57	87.84	97.64
RVA/Pig-tc/USA/Gottfried/1975/G4P[6]	96	90.22	98.74	97.98	98.43	94.61	77.94	89.2	88.43	92.35	98.32
RVA/Pig-tc/USA/OSU/1975/G5P97	75.94	64.62	94.21	98.16	99.21	94.01	82.89	91.82	88.22	93.14	97.81
RVA/Pig-wt/IND/HP113/2002/G6P[13]	74.72	65.34	92.7	99.26	98.31	94.37	97.11	97.17	83.97	89.02	96.3
RVA/Pig-wt/IND/RU172/2002/G12P[7]	72.17	58.547*	94.71	99.82	99.2*	94.25	96.49	86.06	92.36	89.02	98.48
RVA/Pig-tc/CHN/SCJY-11/2017/G9P[23]	74.82	69.23	94.21	96.32	98.2	96.89	76.08	89.94	93.1	89.02	95.79
RVA/Pig-wt/KOR/PRG921/2006/G9[P23]	75.03	70.23	93.44	96.78	99.1	96.65	75.46	88.57	93.42	92.35	97.81
RVA/Pig-wt/KOR/PRG9235/2006/G9P[23]	74.92	70.14	93.95	97.06	99.21	96.77	77.32	86.16	93.42	92.35	97.64
RVA/Pig-wt/CHN/LNCY/2016/G3P[13]	74.92	65.61	92.19	97.18	98.99	96.89	76.91	88.05	93.84	87.84	96.13
RVA/Pig-wt/JPN/BU8/2014/G4P[6]	85.22	84.52	94.46	97.43	99.33	94.73	75.67	87.32	93.95	92.55	88.05
RVA/Human-wt/THA/CMH-N016-10/2010/G4P[6]	86.95	85.52	98.74	97.24	99.21	94.85	76.7	89.2	91.83	88.82	98.65
RVA/Human-wt/KEN/KCH148/2019/G4P[6]	85.83	85.88	99.5	96.51	98.76	93.89	92.37	89.1	83.86	88.43	96.46
RVA/Human-wt/IND/mani-362/2007/G4P[6]	86.03	85*	96.47	97.69*	93.72*	98*	76.7	90.57	87.37	88.43	98.48
RVA/Human-wt/COD/KisB332/2008/G4P[6]	95.82	84.89	98.24	96.05	97.84	93.64	82.68	88.99	84.08	89.22	97.64
RVA/Human-wt/CHN/E931/2008/G4P[6]	87.36	85.88	98.74	98.9	99.55	93.41	77.53	87.84	87.15	87.65	96.63
RVA/Human-wt/CHN/Gx77/2010/G4P[6]	87.26	86.06	99.5	98.35	98.31	97.84	75.88	88.05	87.58	87.45	96.3
RVA/Human-wt/PRY/1809SR/2009/G4P[6]	90.46	85.61	99.5	97.79	99.44	94.85	78.76	88.16	83.76	90.59	94.95
RVA/Human-tc/CHN/R479/2004/G4P[6]	93.85	93.21	94.21	98.9	97.98	97.37	92.99	X	84.82	89.02	96.63
RVA/Human-wt/HUN/BP1547/2005/G4P[6]	91.69	84.79	94.21	96.88	98.43	93.77	76.49	88.38	83.55	89.02	96.63
RVA/Human-wt/RUS/Novosibirsk/Nov11-N2687/2011/G4P[6]	90.77	94.02	99.75	X	X	X	X	87.21	X	X	95.96
RVA/Human-wt/IND/CMC_00038/2011/G4PX	98.77	X	99.24	98.9	99.21	93.79	X	96.89	92.14	96.27	98.48
RVA/Human-wt/IND/NIV929893/1992/G1P[19]	76.99	75.84	99.24	X	X	X	X	X	X	96.86	X

“X” indicates that the sequence data is not available in the NCBI Gene bank and study sequence (NIV1740786); “*” Indicates the sequence is partial in the NCBI Gene bank and study sequence (NIV1740786).

The maximum and minimum identity of gene are colour coded as green and red, respectively.

### 3.3 Picornavirus

**Figure 3:**
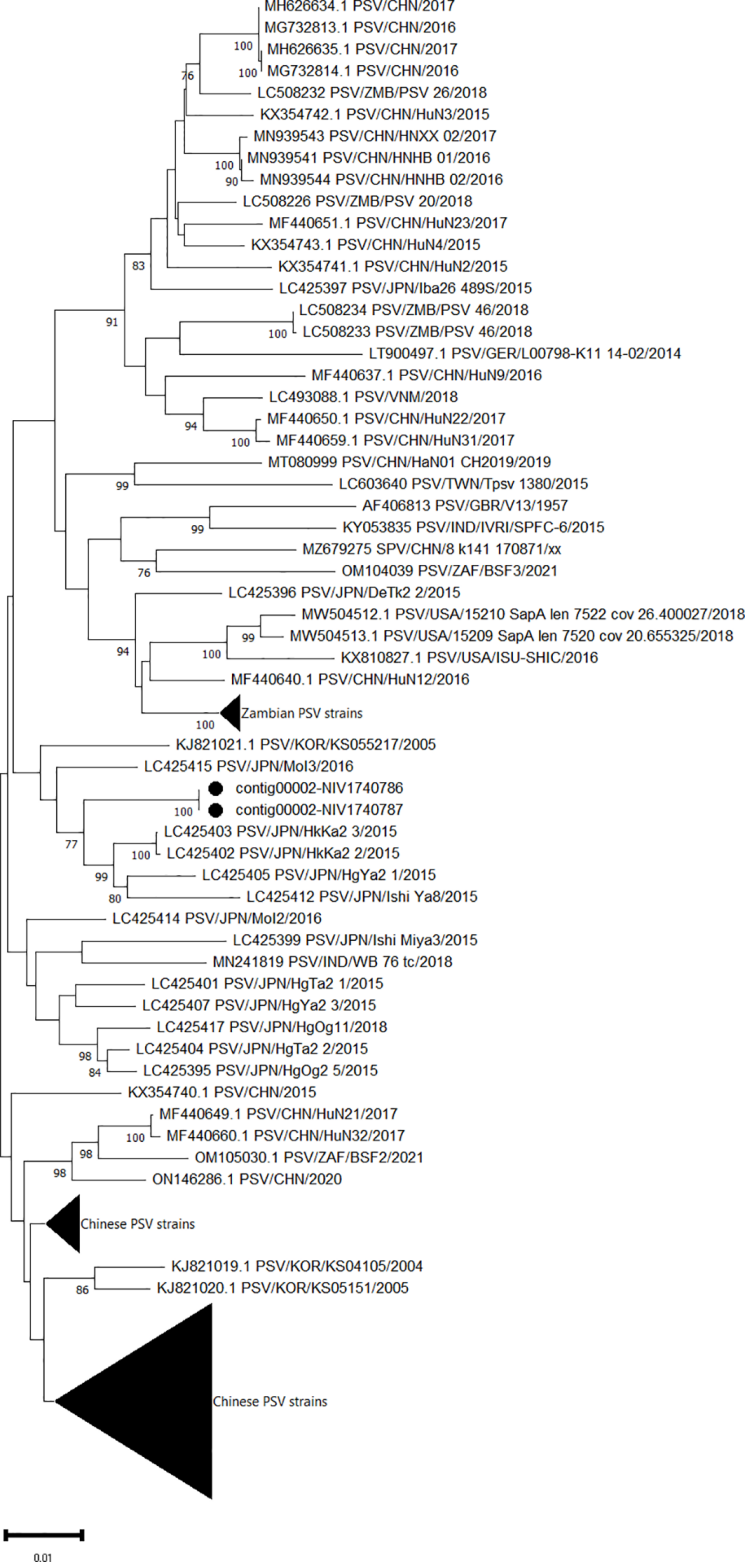
The phylogenetic tree based on amino acid sequences of the sapelovirus polyprotein: The neighbour-joining tree was established using the pairwise distance model with 1000 bootstrap replicates in MEGA 11.0 software. The bar represents the genetic distance while numbers indicate the bootstrap replicates (>75% are shown at branch points). The detected strains are marked with a black circle.

Eight genomes belonging to the family *Picornaviridae* were assembled. Out of which, two identical complete ORFs encoding a single polyprotein of 2323 AA were obtained for PSV (contig00002-NIV1740786 & NIV1740787). The detected PSV strain has a maximum 97.5% AA identity to the Japanese HkKa2-3 and HkKa2-2 strains (**Figure 3**), while it was distantly related to only two available full genomes of Indian WB_76_tc (94.05%) and SPFC-6 (93.31%) strains. Although both study strains didn’t show insertions or deletions at the 3’ end of VP1 as shown by multiple sapelovirus strains, the mutation at the 898-900 AA site from PAT to TAE was detected in comparison to the prototype strain (UK/V13).

**Figure 4:**
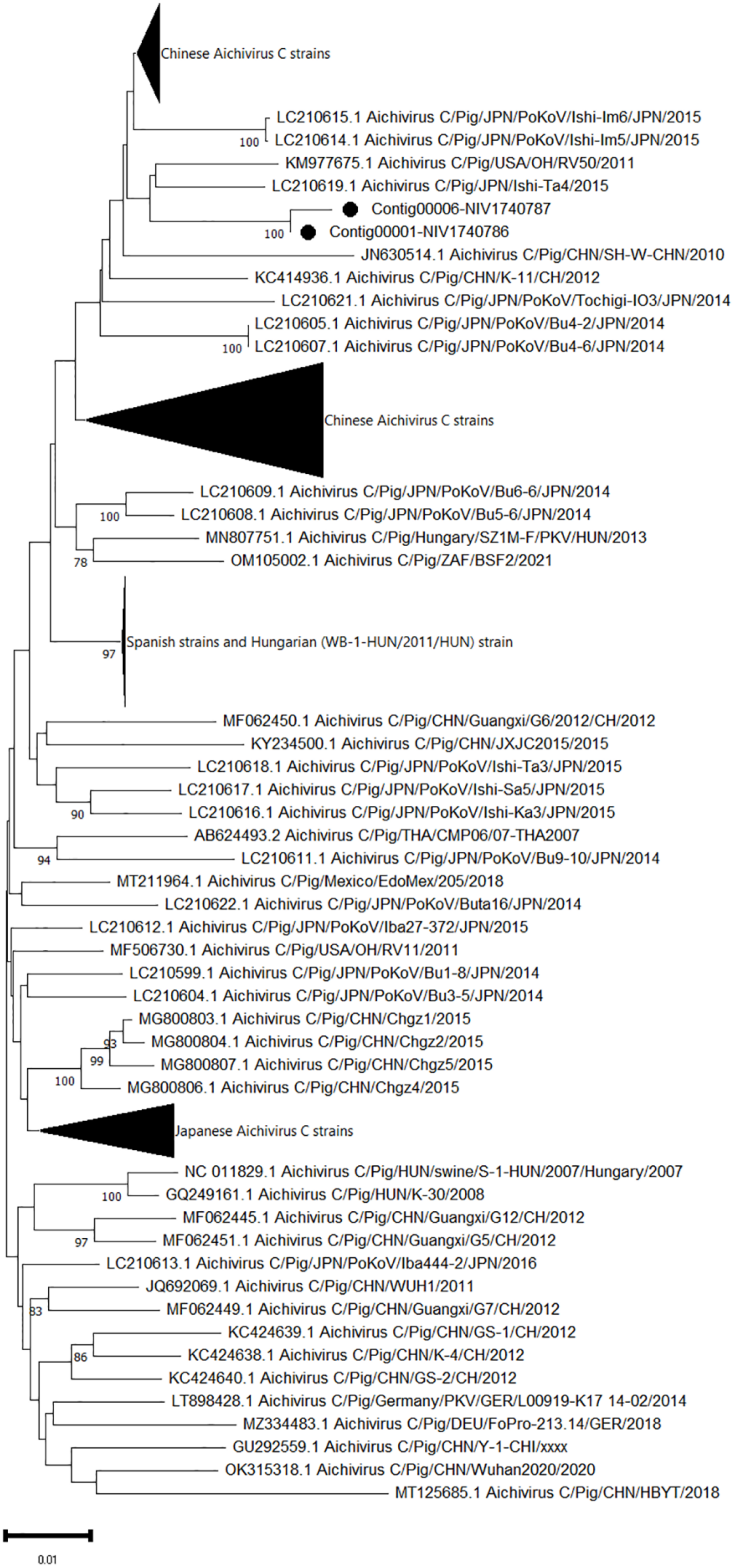
The phylogenetic tree based on amino acid sequences of porcine aichivirus C polyprotein: The neighbour-joining tree was established using the pairwise distance model with 1000 bootstrap replicates in MEGA 11.0 software. The bar represents the genetic distance, while numbers indicate the bootstrap replicates (>75% are shown at branch points). The detected strains are marked with a black circle.

One complete (contig00001-NIV1740786) and one partial sequence (contig00006-NIV1740787) of ORF coding for polyprotein 2459 AA and 1537AA, respectively, of Aichivirus C was retrieved. At the amino acid level, both contigs were 99.7% identical. The amino acid identity between the contig00001-NIV1740786 and the complete polyprotein gene sequences available in the NCBI database varied from 99.93 to 99.97%, with a maximum of 99.0% for the American OH/RV50/2011 and Japanese Ishi-Ta4 strains and the lowest identity for the swine/HBYT/2018/China strain (**Figure 4**). The partial sequence contig00006-NIV1740787 was 96.7% identical to the above strains.

**Figure 5:**
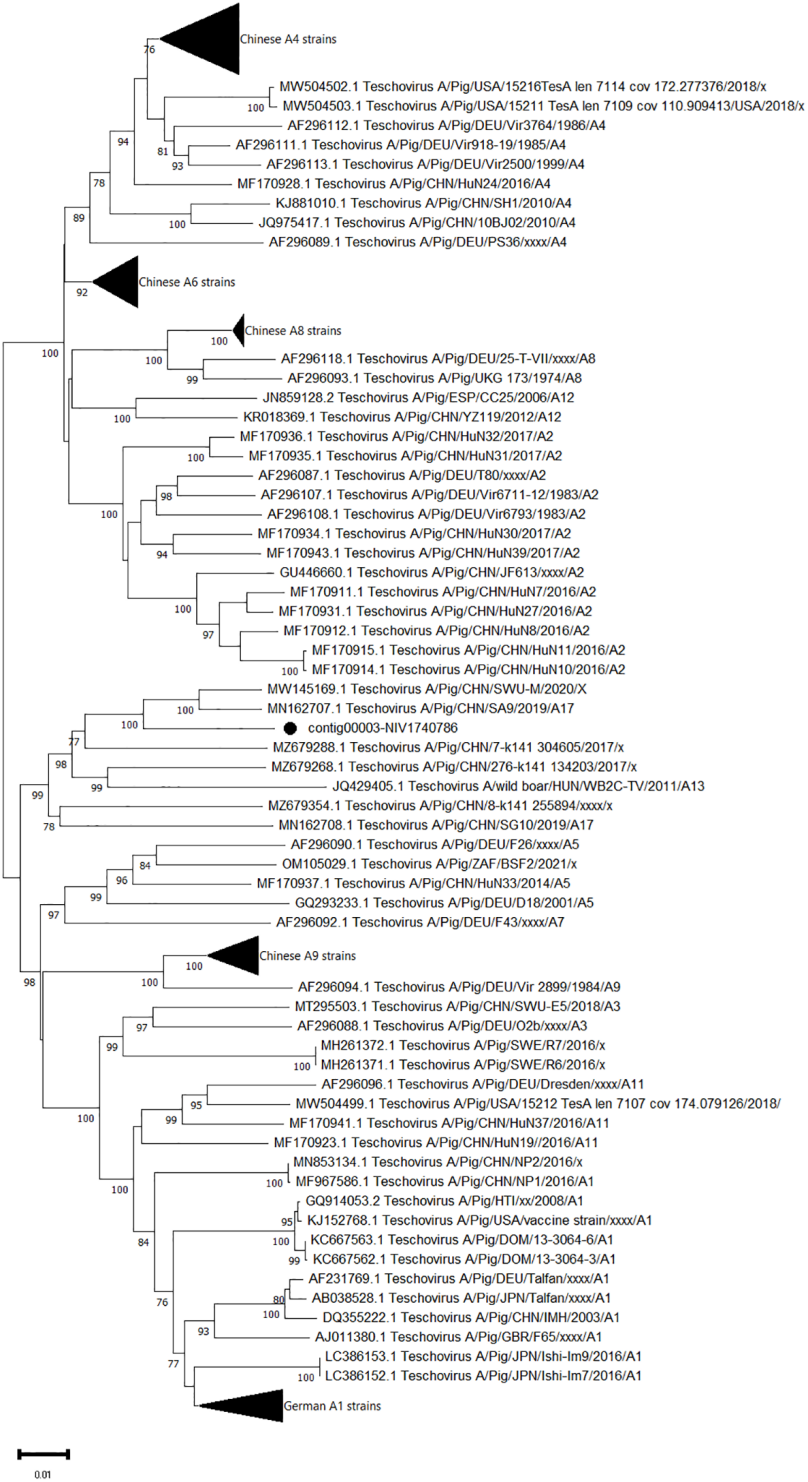
The phylogenetic tree based on amino acid sequences of porcine teschovirus polyprotein: The neighbour-joining tree was established using the pairwise distance model with 1000 bootstrap replicates in MEGA 11.0 software. The bar represents the genetic distance, while numbers indicate the bootstrap replicates (>75% are shown at branch points). The detected strains are marked with a black circle.

The complete sequences of polyprotein genes consisting of 2238 AA were obtained for one PTV strain (contig00003-NIV1740786). It exhibited 94.7-95% AA identity to the Chinese SA9 (PTV-17) and SWU-M strains (**Figure 5**). The VP1 gene of the study strain, when aligned with the prototype and proposed genotype sequences, showed 90.1-90.8% AA identity with PTV-17 Chinese SA9 and PTV-China/SWU-M/2020 strains.

The protease cleavage sites predicted after alignment with other picornaviruses revealed that the polyproteins of all the detected picornaviruses could be processed into mature peptides: L-VP0-VP3-VP1-2A-2B-2C-3A-3B-3C-3D. The ORF regions were flanked by partial 5’UTR and 3’UTR in all the viruses, which were not included in the analysis. The most present putative cleavage site was Gln-Gly (Gln-Gly, Q/G), except for VP0/VP3 (Gln-His, Q/H), VP3/VP1 (Gln-Ala, Q/A), VP1/2A (Gln-Cys, Q/C) in Aichivirus C, VP1/2A (Gln-Leu, Q/L) in PSV, and 2A/2B (Gly-Pro, G/P) in PTV.

### 3.4 Astrovirus

**Figure 6:**
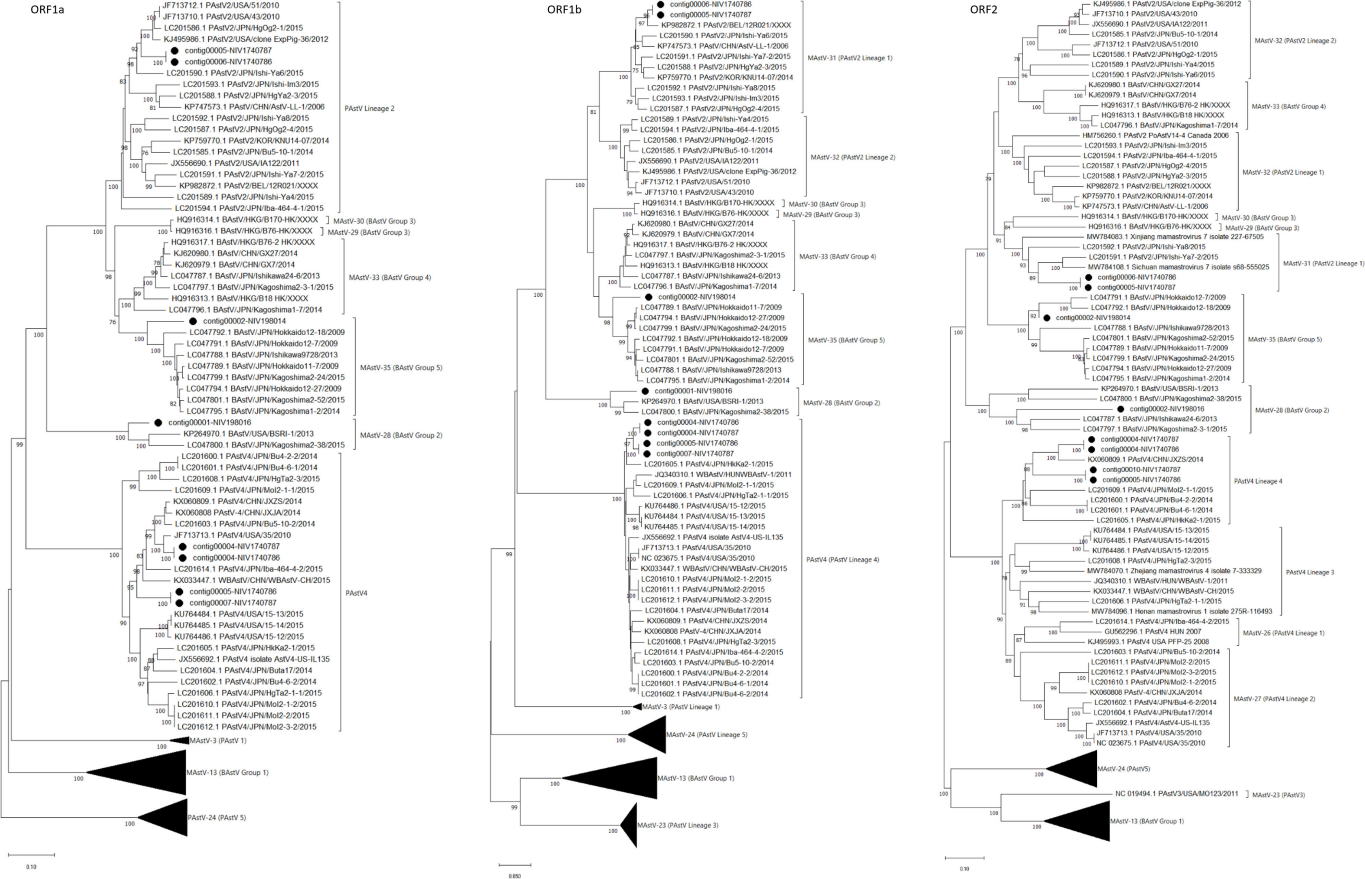
The phylogenetic tree based on amino acid sequences of porcine and bovine astrovirus ORF1a, ORF1b, and ORF2 proteins: The neighbour-joining tree was established using the pairwise distance model with 1000 bootstrap replicates in MEGA 11.0 software. The bar represents the genetic distance, while numbers indicate the bootstrap replicates (>75% are shown at branch points). The lineages to PAstV and Groups to BAstV were assigned according to previous reports (Ito et al., 2017; Zhu et al., 2021). The detected strains are marked with a black circle.

The reads aligned with astroviruses assembled into three nearly complete genomes in each sample. Considering the higher genetic variability in capsid protein (ORF2), phylogenetic analysis and pairwise amino acid identity comparison were performed to demarcate the AstV (**Table 3**; **Figure 6**). Out of six ORF sequences, four and two sequences belonged to the PAstV4 and PAstV2 genotypes, respectively. Previously, the PAstV4 strains were assigned to PAstV4 lineages 1 to 4 (PAstV4 L1-L4) and PAstV2 strains to two lineages 1 to 2 (PAstV2 L1-L2) (Ito et al., 2017). The porcine study strains (contig 00004-NIV1740786 & NIV1740787; contig00005-NIV1740786, contig00010-NIV1740787) were 74.7% identical and belonged to the PAstV4 L4 lineage. The identical contig00004-NIV1740786 & NIV1740787 showed a maximum AA identity of 86.6%, while identical contigs00005-NIV1740786 & contig00010-NIV1740787 exhibited the highest identity of 71.3% with the Chinese JXZS strain that formed a group with Japanese PAstV strains. Another pair of identical PAstV (contig00006-NIV1740786 & contig00005-NIV1740787) strains showed a maximum AA identity of 75.6-76.9% with Japanese porcine Ishi-Ya7-2, Ishi-Y8, and Sichuan astrovirus s68-555025 strains and belonged to the PAstV2 L1 lineage containing Japanese, Chinese, Korean, Belgian, and Canadian PAstV2 strains, and this cluster was previously reported as proposed MAstV-31 (Ito et al., 2017). The identified PAstV2 strains shared a branch with closely related BAstV strains, Chinese B76-HK (MAstV-29) and B170-HK (MAstV-30).

**Table 3:** The heatmap showing comparative amino acid identities (%) of detected PAstV and BAstV with reference AstVs.

AstV Reference strains	PAstV4						PAstV2			BAstV					
										Group 2			Group 5		
	contig00004-NIV1740786 & contig00004-NIV1740787 (ORF1ab-2)			contig00005-NIV1740786 (ORF1ab-2) contig00007-NIV1740787 (ORF1b) contig00010-NIV1740787 (ORF2)			contig00006-NIV1740786 & contig00005-NIV1740787 (ORF1ab-2)			contig00001-NIV198016 (ORF1ab-2) contig00002-NIV198016 (ORF2)			contig00002-NIV198014 (ORF1ab-2)		
	ORF1a	ORF1b	ORF2	ORF1a	ORF1b	ORF2	ORF1a	ORF1b	ORF2	ORF1a*	ORF1b	ORF2*	ORF1a	ORF1b	ORF2*
PAstV4-CH/JXZS/2014	92.46	95.62	86.67	86.34	95.62	71.46	34.22	60.39	31.09	37.87	62.15	24.06	34.36	57.65	45.74
PAstV4-35/USA	96.12	95.34	52.27	84.57	95.07	51.17	33.93	61.5	28.49	38.02	62.43	22.57	34.57	58.72	39.43
PAstV2-s68-555025	35.39	61.22	32.18	35.27	61.5	30.57	83.01	95.7	76.75	53.19	69.39	28.48	68.38	81.63	64.78
PAstV2-Ishi-Ya7-2	34.16	61.22	33.23	34.53	61.5	32.1	79.61	94.89	76.88	51.47	70.16	27.98	68.01	81.63	63.52
PAstV2-Ishi-Y8	34.77	60.94	30.39	34.65	61.22	29.72	80.95	93.82	75.75	52.7	69.13	30.24	67.89	82.69	64.58
PAstV2-7/227-67505	34.4	60.39	30.31	34.4	60.66	28.97	94.17	91.13	67.9	50.98	69.89	29.37	69.12	83.75	68.24
PAstV2-Bel-12R021	33.74	59.93	32.15	34.11	60.64	30.7	80.07	97.54	56.99	51.23	72.18	30.07	67.98	81.27	66.27
PAstV2-43/USA	34.4	60.11	32.5	34.9	60.11	32.68	90.41	86.83	50.75	51.47	70.43	29.58	69.12	84.1	59.62
PAstV3-US-MO123	23.6	52.88	21.33	23.66	53.57	22.11	24.71	53.91	19.35	25.71	51.98	14.95	26.18	53.9	29.34
PAstV5-33/USA	18.52	49.16	24.65	18.27	48.6	24.23	19.55	50.14	24.35	20.05	49.87	17.45	20.33	49.46	33.54
BAstV/Group2- BSRI-1	33.29	62.43	27.11	33.1	62.43	28.01	44.73	70.16	32.78	84.88	91.1	46.06	44.99	68.55	43.71
BAstV/Group2-Kagoshima2-38	32.9	62.43	29.16	33.38	61.88	29.22	45.01	70.7	33.1	82.44	91.36	41.74	43.82	68.55	45.45
BAstV/Group3-B76-HK	31.88	63.83	31.98	32.88	63.48	31.89	71.48	80.99	57.88	49.38	71.48	33.33	72.77	85.87	68.15
BAstV/Group 5-Hokkaido12-18	33.29	60.99	33.14	34.41	60.99	33.48	68.02	79.58	48.29	49.5	71.83	30.61	83.95	91.52	89.05
BAstV/Group1-USA/NeuroS1	24.05	48.77	21.96	24.23	49.82	22.87	24.21	51.24	20.4	24.61	49.3	16.05	22.69	50.35	29.91
BAstV/Group4-B76-2 HK	33.66	62.06	32.41	34.65	62.41	31.35	72.67	81.69	50.26	50.99	70.07	33.12	75.34	86.93	62.76

“*” Indicates the sequence is partial.

The maximum and minimum identity of gene are colour coded as green and red, respectively.

The RdRp genes of identified PAstV4 genotypes, contig00004-NIV1740786 & NIV1740787, contig00005-NIV1740786, & contig00007-NIV1740787 had 98.1% AA identities (**Table 3**; **Figure 6**). These strains exhibited 94.0-95.6% AA identity with porcine Japanese strains (HkKa2-1, MoI2-1-2, MoI2-3-2, MoI2-2, MoI2-1-1, Bu4-2-2, Bu4-6-1, Bu4-6-2, Bu4-2-2, Iba464-4-2, Bu5-10-2, Buta17, HgTa2-3), Chinese strains (WBAstV-CH, JXZS & JXJA), American strains (35, 15-12, 15-13, 15-14, IL135), and Hungarian (WBAstV-1) PAstV-4 strains. In ORF1a region, the contig00004-NIV1740786 & NIV1740787, contig00005-NIV1740786 & 00007-NIV1740787 shared 84.1% identity (**Table 3**; **Figure 6**). These strains exhibited 79.5-87.2% AA identity with all the above-mentioned strains except Hungarian strain WBAstV-1 (38.5%). The PAstV2 genotype RdRp genes (contig00006-NIV1740786 & contig00005-NIV1740787) were identical. They showed 93.8-97.5% identity with PAstV2 Belgian (12R021), Japanese (Ishi-Ya7-2, HgYa2-3, Ishi-Ya8, HgOg2-4, Ishi-Im3, Ishi-Ya6), Korean (KNU1407), and Chinese (LL-1) strains. ORF1a (contig00006-NIV1740786 & contig00005-NIV1740787) showed a maximum AA identity of 89.9-90.4% with American (43 & 51 strains), Japanese (HgOg2-1), and belonged to the PAstV2 lineage. It was therefore evident that there were PAstV4 & PAstV2 lineage viruses within the stool samples from both piglets, with two different capsids, ORF1a and ORF1b, sequences present.

In bovine samples, AstV was the predominant virus, with a recovery of 112 contigs (NIV198014) and 524 contigs (NIV198016). Out of these, contigs covering ORF1a (contig00002-NIV198014, contig00001-NIV198016), ORF1b (contig00002-NIV198014, contig00001-NIV198016), and ORF2 (contig00002-NIV198014, contig00002-NIV198016) were included, while all the partial contigs were excluded from the analysis (**Table 3**; **Figure 6**). The bovine RdRp gene of contig00001-NIV198016 and contig00002-NIV198014 shared a 70.7% AA identity. The complete RdRp and partial ORF1a (410 AA) regions of contig00001-NIV198016 and partial 482 AA (ORF2) of contig00002-NIV198016 showed identity in a range of 91.1-91.4% and 81.2-84.1% with bovine Japanese Kagoshima2-38 and American BSRI-1 strains, and 61.9% identity with Japanese Ishikawa24-6 belonging to BAstV group 2 which corresponds to MAstV-28.

The contig00002-NIV198014 exhibited 91.2-91.9% in the RdRp region, 82.7-83.8% in the ORF1a region, and 82.5-90.8% in the partial ORF2 (338 AA long) region with BAstV Group 5 consisting of Japanese strains, which corresponds to the proposed MAstV-35. One partial 410-AA-long ORF1a sequence (contig00003-NIV198014) from the same sample shared 82.8-88.6% AA identity with Group 4 (MAstV-33). The 198 AA partial ORF1b of contig00009-NIV198014 shared 99.0% AA identity with the Japanese Kagoshima 2-3-1 strain, the strain of which ORF1a & ORF1b clustered with group 4 and ORF2 with group 2 without evidence of a recombination event (Nagai et al., 2015). The 302-AA-long partial ORF2 sequence of contig00006-NIV198014, contig00011-NIV198014 (192 AA) & contig00012-NIV198014 (181 AA), as well as contig00005-NIV198014 ORF1a (244 AA), were excluded as they were non-overlapping with reference sequences included in phylogenetic tree construction.

### 3.5 Recombination analysis

**Figure 7:**
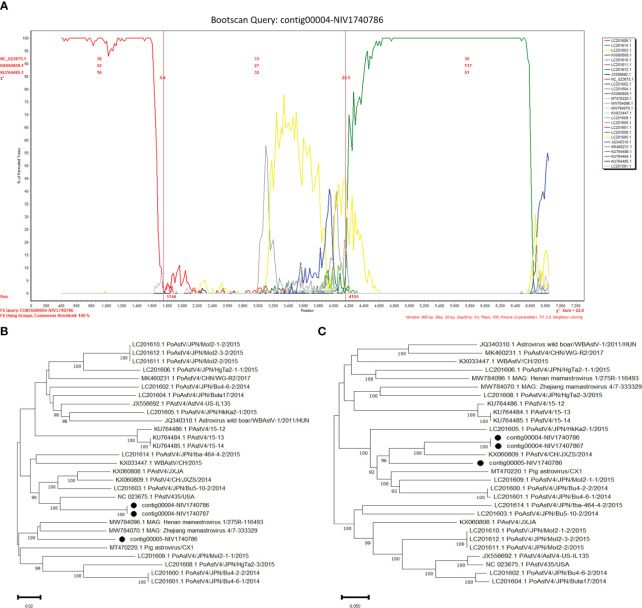
Recombination analysis of Indian PAstV4 (contig00004-NIV1740786 & NIV1740787): The bootscan analysis was performed with 800bp window size, 10bp step size, 100 bootstrap replicates, gap-stripped alignments, and neighbour-joining algorithm. The identified strains are marked with black dots. **(A)** The bootscan plots depicting Indian PAstV4 and parent strains: 35/USA strain (NC_023675) and CH/JXZS/2014 strain (KX060809.1). The phylogenetic trees were constructed using the Neighbour Joining method based on the **(B)** 1-4155 and **(C)** 4156-6910 nt region of the PAstV-4 strain.

**Figure 8:**
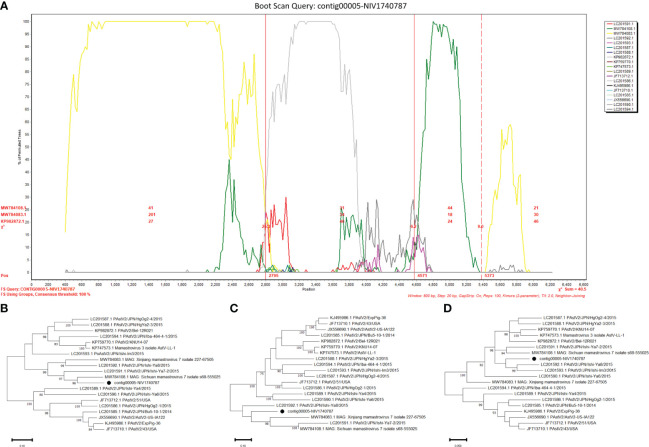
Recombination analysis of Indian PAstV2 (contig00005-NIV1740787): The bootscan analysis was performed with 800bp window size, 10bp step size, 100 bootstrap replicates, gap-stripped alignments and neighbour-joining algorithm. The identified strains are marked with black dots. **(A)** The bootscan plots depict Indian PAstV2 and parent strains: 227-67505 strain (MW784083.1), Bel-12R021 strain (KP982872.1), and s68-555025 strain (MW784108.1). The phylogenetic trees were constructed using the Neighbour Joining method based on the **(B)** 1-2795, **(C)** 2796-4571 and, **(D)** 4572-5373 nt regions of the PAstV-2 strain.

The potential recombinant events were identified in the PAstV4 and PAstV2. The PAstV4 strains (contig00004-NIV1740786 & NIV1740787) were recombinants arising from strains PoAstV4/35/USA/2010 and PoAstV4/CH/JXZS/2014 (**Figure 7A**). The boot scan plot observed a breakpoint at nucleotide (nt) position 4155. The phylogenetic trees showed that before the breakpoint, PAstV4 study strains clustered with the 35/USA strain, whereas after the breakpoint they clustered with the JXZS strain (**Figures 7B, C**). The PAstV2 (contig00005-NIV1740787) strain was recombinant with Xinjiang mamastrovirus 7/227-67505, PoAstV2/Bel-12R021, and PoAstV2/Sichuan mamastrovirus 7/isolate s68-555025 (**Figure 8A**). The three breakpoints were visible at 2795, 4571, and 5373 nt positions. To confirm the results, phylogenetic trees were constructed using all the fragments of the recombinant virus (**Figures 8B, D**). The PAstV2 study strain was grouped with the 227-67505 strain before 2795, the Bel-12R021 strain between 2795-4571, and the s68-555025 strain between 4571-5373 fragments. The above observations indicated that the PAstV4 strain (contig00004-NIV1740786) and PAstV2 strain (contig00005-NIV1740787) might be a potent recombinant. In the other detected viruses evidence of recombination was not detected.

## 4 Discussion

Diarrhoea has multiple etiologies and causes huge economic losses to cattle and pig farmers worldwide. Traditionally, infectious causes of diarrhoea were diagnosed as single etiologies. However, with next-generation sequencing, the evidence indicates that many microbes are present in the faeces of animals. The present metatranscriptome study of RVA-positive porcine diarrheic faecal samples revealed the presence of diverse RNA viruses, including picornavirus and AstV, in piglets, while only AstV was found in bovine samples.

The G4P[6] genotype combination in piglets is common but considered unusual in humans (Papp et al., 2013b; Zhou et al., 2015). Although the G4P[6] study strains resembled the prototype Gottfried genotype constellation, the phylogenetic analysis revealed that the VP7 gene was more similar to those of human strains in G4 lineage-1, indicating RVA genome reassortment during coinfection and interspecies transmission. However, such anthrapozoontic and zooanthraponotic transmission of G4P[6] strains with or without reassortment might be in place for quite a long time in India (Mukherjee et al., 2011; Giri et al., 2019). The VP6 gene was considered of porcine origin although it shared the highest nucleotide identity (94.90- 97.57%) with Indian human (RV1020, CMC_00038, NIV929893) strains because: i) the VP6 gene shared the highest nucleotide identity (96.15-96.73%) with Indian porcine (UP-Por30, UP-Por34) strains ii) The VP6 gene shared a low nucleotide identity (90.20-90.79%) with Wa strain (G1P[8]). These outcomes support the previous observations about the potential porcine origin of the I1 VP6 gene in sporadic human strains, and it is more common in pigs (Papp et al., 2013b; Zhou et al., 2015). The low nucleotide and amino acid identities were observed for the VP4 and NSP3 genes. This reflects the lack of known close relatives to the study strain because of low surveillance and the full genome characterisation of porcine RVAs. The constant infections of G4P[6] strains in humans necessitate their surveillance in both humans and porcine to understand human-to-human transmission and its impact on the effectiveness of introduced vaccines.

In addition to the RVA, the study detected different RNA viruses like PAstV, enterovirus G, PSV, Aichivirus C, and PTV in pigs with diarrhoea but their number is mostly single, except AstV. The detection of multiple viruses in diarrheic piglets in the present study indicates that infection by one virus might be predisposed to infection by other viruses. This may be because of the capacity of rotaviruses to sustain the initial infection; rotaviruses are more adept at replicating in the host; and interference by rotaviruses leads to infection by other members of the virome (Doerksen et al., 2022).

The highest genera (4 out of 6) detected in the present study belong to the family *Picornaviridae*. The detection of diverse picornaviruses in pig stools is not unusual, as such coinfections have already been reported (Shan et al., 2011; Chen et al., 2018). There are multiple reasons for detecting picornaviruses compared to other enteric viruses in clinical samples. Among them, their excretion in large numbers, resistance to the environment, which increases survivability, and longer exposure periods for pigs increase their chances of detection. On one hand, the above features make them easier to detect, but on the other, they increase the bias of their detection in mixed infections. The present study has not only detected mixed infections but also recovered the full genome sequences of PAstV, EVG, PSV, Aichivirus C, and PTV, genetically characterizing them. The clinical significance of all the detected viruses is not elucidated, as they are detected in healthy and diseased animals. However, the complete genome sequences of the above viruses will facilitate the development of their diagnostics and the design of future epidemiological studies.

Among the porcine AstVs, PAstV1-5, PAstV4 is reported to have the highest prevalence in domestic pigs from the USA, Europe, and Asia (Folgueiras-González et al., 2021). In the Indian pig population, all five PAstV lineages are in circulation, with a predominance of lineage 4, followed by lineage 2 (Kattoor et al., 2019; Kour et al., 2021). Earlier Indian studies amplified partial ORF1a and/or ORF2 genome regions; as a result, only partial PAstV sequences are available. However, the present study, employing a metatranscriptomic approach for the first time, contributed to the recovery of two whole genome sequences from Indian pigs. Overall, the present study detected five strains of the PAstV4 lineage and two strains of the PAstV2 lineage, representing a diversity of viruses in Indian pigs; however, the study aims not to investigate the prevalence of the virus. In the phylogenetic tree, PAstV4 study strains were part of the PAstV4 L4 lineage, and PAstV2 strains were part of the PAstV2 L1 lineage, consisting of Japanese and Chinese strains. However, the origins of AstV strains could not be elucidated as most of the available sequences in GenBank are from the USA, Japan, and China. RNA recombination is a driving force in the evolution, emergence, and virulence of AstVs (Zhu et al., 2021). The parents of the PAstV4 recombinant strain were identified from the USA and China (Jiangxi), whereas three parental strains of the PAstV2 recombinant strain were from Belgium and two provinces of China (Xinjiang and Sichuan). The recombination in AstV indicated that the pig trade and faecal-oral transmission promoted the recombination between strains from geographically different locations.

Neonatal calf diarrhoea is most commonly caused by RVA, and its co-infection has been reported with BAstV in Korea (Oem and An, 2014), China (Alfred et al., 2015; Zhu et al., 2021), and Italy (Martella et al., 2020). Although previous studies could not establish an association of AstV with diarrhoea, a recent metagenomics study found most of the BAstV reads in a diarrheic compared to the healthy Chinese calves (Lu et al., 2021). Another study on Chinese calves established a positive correlation between the presence of only BAstV and co-infection in diarrhoea (Zhu et al., 2022). In concurrence, the present study first time recovered BAstV suggesting that the major viral pathogen associated with calf diarrhoea might be AstV. However, the lack of complete genome sequences makes it difficult to explore the precise origin of the virus and the dynamics of interspecies transmission and zoonotic transmission. In addition to AstV, RVA reads were detected but contigs were not assembled; this may be because of the lower virus load in the samples, which was picked up by the RT-PCR, which has more sensitivity than NGS.

The validation of detected viruses was performed by virus isolation for porcine RVA until the fourth passage in MA104 cells, confirmed by sandwich ELISA and real-time quantitative RT-PCR (unpublished data, **Supplementary File 2**). Moreover, bovine RVA (Sawant et al., 2020a), astrovirus (Sawant et al., 2023), porcine enterovirus G, and porcine teschoviruses (Sawant et al., 2020b) were detected by RT-PCR using specific primers. Nevertheless, the present exploratory study has significant limitations. First, only a few samples- two from bovine and porcine species from the same farms were analysed, resulting in the retrieval of identical viruses, thus restricting exploration of the diversity. The larger and broader sampling will likely demonstrate the temporal dynamics of microbial species. Second, healthy animals were not included in the baseline virome, which would have helped to delineate the changes in RNA virome after RVA infection. Thirdly, the study focused on only RVA-infected diarrhoea; hence, the role of viruses detected in disease could not be delineated. Lastly, validation by isolation of all the viruses was not possible because enteric viruses are difficult to isolate in cell lines, insufficient amount of samples, and cell lines supporting the growth of many detected viruses were not easily available. Nevertheless, the exploration of virome within a sample signifies the advent of a new age in diagnosis, which can discern unknown causes of diarrhoea and provide potent technical support for preventing and controlling diarrhoea.

In summary, the study revealed a diversity of RNA virome in pigs while predominant astrovirus infection in calves with diarrhoea. The results emphasized that RVA-infected pigs are co-infected with diverse RNA viruses, favouring the emergence of new reassortant and recombinant viruses like RVA with the G4P[6] genotype and PAstV, respectively. In the future, such studies with large sample sizes covering different health conditions will play a seminal role in the surveillance of possible zoonotic pathogens at the human-animal interface.

## Data availability statement

The data presented in the study are deposited in the NCBI Sequence Read Archive, https://www.ncbi.nlm.nih.gov/sra (Accession number: PRJNA941130).

## Ethics statement

The present work was carried out in the project ENV-1701 which was approved by the institutional biosafety committee (NIVIBSC/12.12.2017/02) and the animal ethics committee meeting held on 20th December 2017 of ICMR-NIV, Pune. The studies were conducted in accordance with the local legislation and institutional requirements. Written informed consent was obtained from the owners for the participation of their animals in this study.

## Author contributions

PS: Conceptualization, Data curation, Formal Analysis, Funding acquisition, Investigation, Methodology, Project administration, Resources, Software, Supervision, Validation, Visualization, Writing – original draft, Writing – review & editing. AK: Writing – review & editing, Formal Analysis, Methodology, Software, Visualization. RM: Writing – review & editing, Investigation. RP: Writing – review & editing, Investigation. ML: Writing – review & editing, Supervision.
