# Supplementary material for: Metatranscriptomic assessment of diarrhoeic faeces reveals diverse RNA viruses in rotavirus group A infected piglets and calves from India
Source: Front Cell Infect Microbiol. 2023 Oct 27;13:1258660. doi: 10.3389/fcimb.2023.1258660 (PMC10642067; doi:10.3389/fcimb.2023.1258660)
Supplement: Supplementary file 1 [file DataSheet_1.pdf]

Whole Genome Sequencing & Analysis of Rotavirus

Genotypic Project ID : **SO_9305**

Client details : **Dr Pradeep Sawant**

NIV, Pune, Maharashtra

PREPARED BY:
Mr. Nihar

REVIEWED BY:
Dr. Gandhimathi

Genotypic Technology [P] Ltd., #2/13, Balaji Complex, 80 feet road, R.M.V. 2nd Stage, Bangalore-560094, INDIA

Phone: +91 80 40538245/8202; Fax: +91 80 40538222

Website: www.genotypic.co.in, E-mail: ngsservices@genotypic.co.in

TABLE OF CONTENTS

1. AIM 3

1.1 Project Objectives 3

2. MATERIALS & METHODS 3

2.1 RNA Quality Control 3

2.2 Library Preparation 3

2.3 Illumina Sequencing 5

2.4 Data Analysis 5

3. RESULTS 7

3.1 RNA QC 7

3.2 Illumina Library QC and data de-multiplexing 7

3.3 Primary Analysis 10

3.4 Secondary Analysis 10

3.4.1 Host Contamination Removal 10

3.4.2 Reference Viral Genome based Alignment 11

3.4.3 Variant Prediction & annotation 11

4. SUMMARY 11

5. REFERENCES 12

1. AIM

The aim of the experiment is to perform whole genome sequencing based alignment, variant calling and annotation of 4 viral samples.

1.1 Project Objectives

a) Raw data QC check
b) Alignment of host contamination free processed reads against reference genome
c) Variant prediction & annotation

2. MATERIALS & METHODS

2.1 RNA Quality Control

The quantification and quality of the RNA was assessed using Nanodrop2000 (Thermo Scientific, USA), RNA HSQubit(Thermo Scientific, USA).

2.2 Library Preparation

RNA sequencing libraries were prepared with NEBNext® Ultra™ II Directional RNA Library Prep Kit (New England BioLabs, MA, USA)at Genotypic Technology Pvt. Ltd., Bangalore, India.

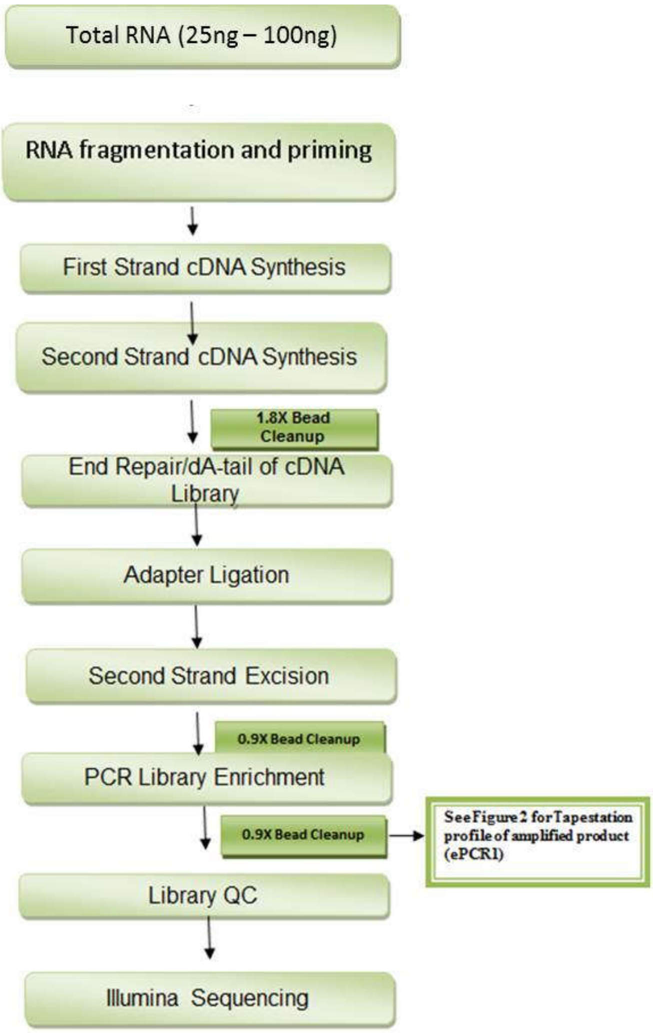

Figure 1: Work flow of NEBNext® Ultra™ II Directional RNA Library Kit

50ng of total RNA was taken for fragmentation and priming. Fragmented and primed RNA was further subjected to first strand synthesis followed by second strand synthesis. The double stranded cDNA was purified using JetSeq Beads (Bioline, Cat # BIO-68031). Purified cDNA was end-repaired, adenylated and ligated to Illumina multiplex barcode adapters as per NEBNext® Ultra™ II Directional RNA Library Prep protocol followed by second strand excision using USER enzyme at 37 ˚C for 15mins.

Illumina Adapters used in the study were:

Universal Adapters

5’AATGATACGGCGACCACCGAGATCTACACTCTTTCCCTACACGACGCTCTTCCG

ATCT-3' and
Index Adapter:
5'-GATCGGAAGAGCACACGTCTGAACTCCAGTCAC [INDEX]
ATCTCGTATGCCGTCTTCTGCTTG-3'.
[INDEX] – Unique sequence to identify sample-specific sequencing data

Adapter ligated cDNA was purified using JetSeq Beads and was subjected to 11 cycles for Indexing-(98˚C for 30 sec, cycling (98˚C for 10sec, 65˚C for 75sec) and 65˚C for 5min) to enrich the adapter-ligated fragments. Final PCR product (sequencing libraries) was purified with JetSeq Beads, followed by library quality control check. Illumina-compatible sequencing libraries were quantified by Qubit fluorometer (Thermo Fisher Scientific, MA, USA) and its fragment size distribution was analyzed on Agilent 2200 Tapestation

2.3 Illumina Sequencing

The libraries were paired-end sequenced on Illumina HiSeq X Ten sequencer (Illumina, San Diego, USA) for 150 bp cycles following manufacturer's instructions.

2.4 Data Analysis

Libraries were sequenced using Illumina Hiseq (150 x 2 chemistry) for 4 viral samples. The low quality reads were trimmed using Trim Galore-v0.4.4[3]. It is a wrapper tool around Cutadapt and FastQC to consistently apply quality and adapter trimming to FastQ files. The adapter clipped, high quality reads from 4 samples were aligned against host genomes (2 samples against porcine as host and 2 samples against waterbuffalo as host) using Bowtie2-v2.2.5[4] alignment tool. Bowtie 2 is an ultrafast and memory-efficient tool for aligning sequencing reads to long reference sequences. It is particularly good at aligning reads of about 50 up to 100s or 1,000s of characters, and particularly good at aligning to relatively long genomes. Alignment pre-processing was performed using Samtools. It is a set of utilities that manipulate alignments in the SAM (Sequence Alignment/Map), BAM, and CRAM formats, which converts between the formats, does sorting, merging and indexing and can retrieve reads in any regions. The unaligned reads against host genomes were filtered out and mapped with reference rota virus genomes (2 samples mapped against porcine rota virus gottfried strain and 2 samples mapped against bovine rota virus NCDV

strain). The samples aligned against bovine rota virus NCDV strain had very poor alignment with few reads mapping, hence excluded from further downstream analysis. The other 2 samples (1740786 & 1740787) aligned against porcine rota virus gottfried strain were used further for variant calling and annotation purpose. The variants were identified from processed alignment data using Samtools-v1.9[5] pipeline. Finally, annotation was carried out for all the identified variants using SnpEff[6], which quickly identify and classify disease-relevant variants, and then communicate significant findings in a structured report. The work flow of the analysis is depicted in Figure 2.

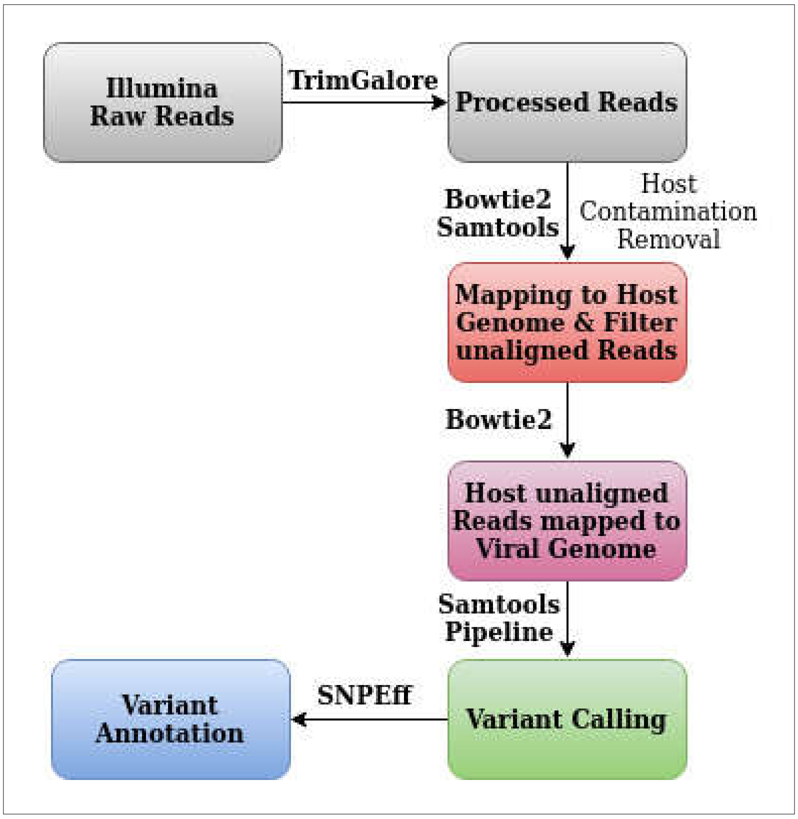

Figure 2: Bioinformatics workflow for WGS analysis

3. RESULTS

3.1 RNA QC

The samples passed quality and quantity assessment with optimal yield and concentration, suitable for Illumina library preparation (Table 1).

Table 1: Estimated RNA concentration and purity

Sl. No.	Sample Name	ng/µl	260/280	260/230	Qubit Conc. (ng/µl)	Volume (µl)	Yield (ng)
1	1740786	293.4	3.5	1.32	10	23	230
2	1740787	312.9	3.5	1	10.5	23	241.5
3	198014	303.5	3.52	1.41	11	23	253
4	198016	283.9	3.52	1.38	11.5	23	264.5

3.2 Illumina Library QC and data de-multiplexing

The Illumina-compatible sequencing library for the sample showed an average fragment size of 410bp, as well as sufficient concentration for obtaining desired sequencing data. The data obtained from the sequencing run was demultiplexed using Bcl2fastq software v2.20 and FastQ files were generated based on the unique dual barcode sequences. The sequencing quality was assessed using FastQC v0.11.8 software. The adapter sequences were trimmed and bases above Q30 were considered and low quality bases were filtered off during read pre-processing and used for downstream analysis (Table 2 and Figure 3a-3d).

Table 2: Description of the libraries

S.No.	Sample ID	Qubit (ng/µl)	Vol (µl)	Yield (ng)	Index	Index Sequence
1	SO_9305_1740786	4.56	10	45.6	NEB09	GATCAG
2	SO_9305_1740787	22.8	10	228	NEB10	TAGCTT
3	SO_9305_198014	5.48	10	54.8	NEB11	GGCTAC
4	SO_9305_198016	6.4	10	64	NEB12	CTTGTA

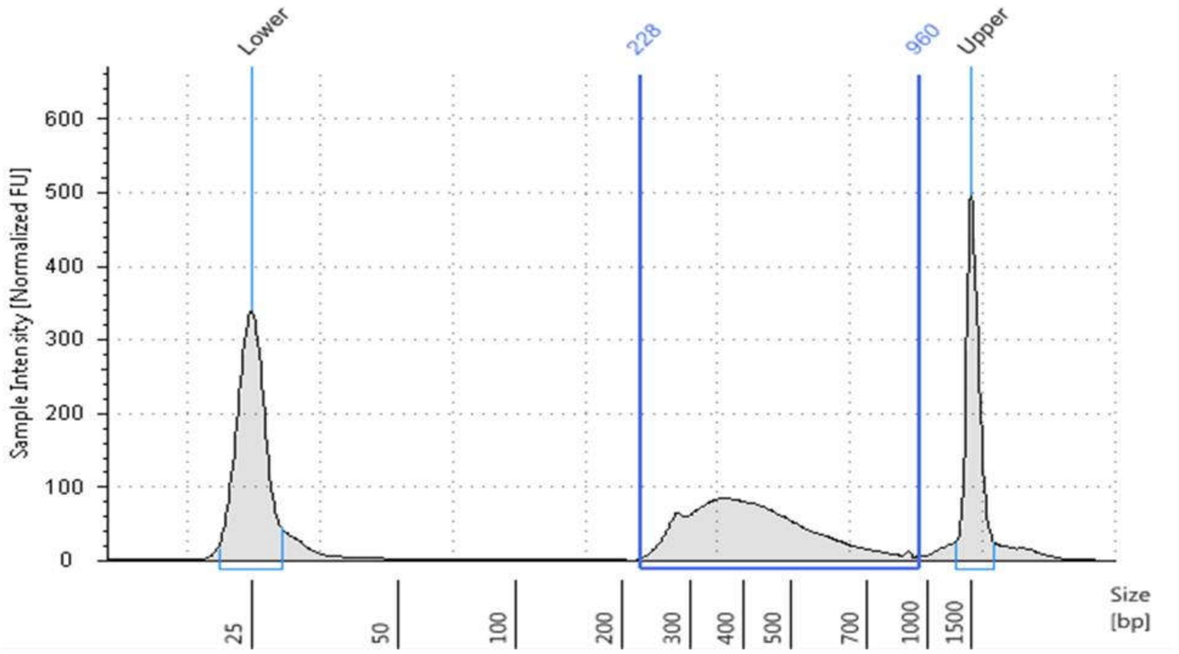

From [bp]	To [bp]	Average Size [bp]	Conc. [pg/µl]	Region Molarity [pm	% of Total
228	960	432	401	1570	81.48

Figure 3a: Tape Station profile of 1740786

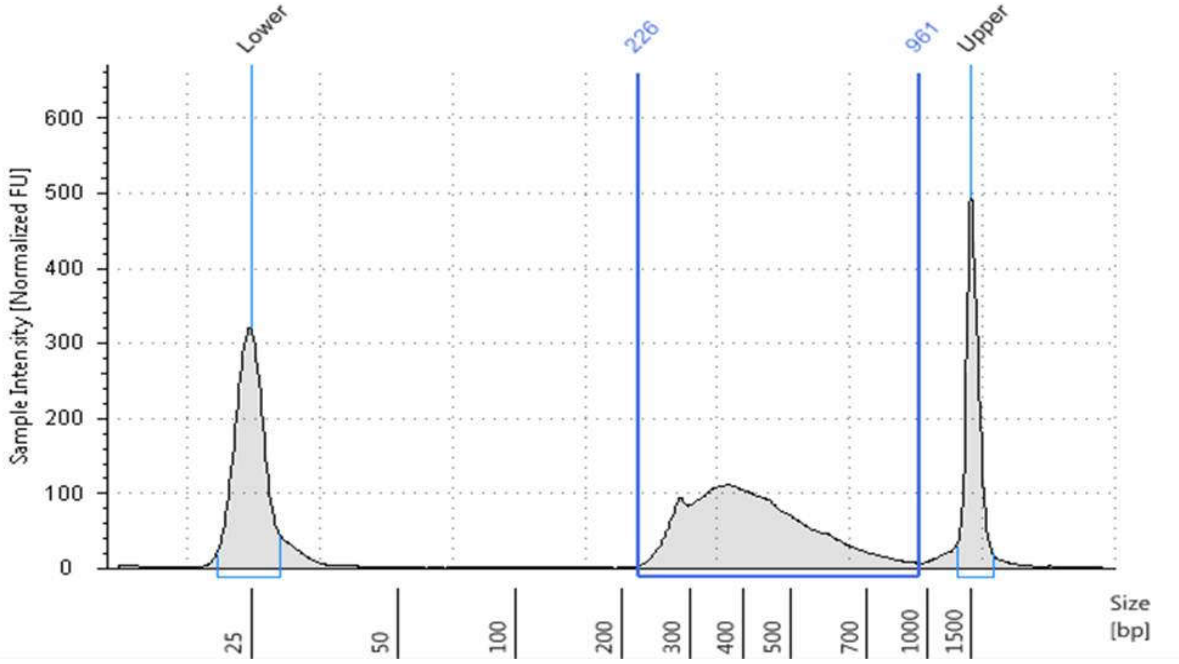

From [bp]	To [bp]	Average Size [bp]	Conc. [pg/µl]	Region Molarity [pm	% of Total
226	961	434	552	2130	88.48

Figure 3b: Tape Station profile of 1740787

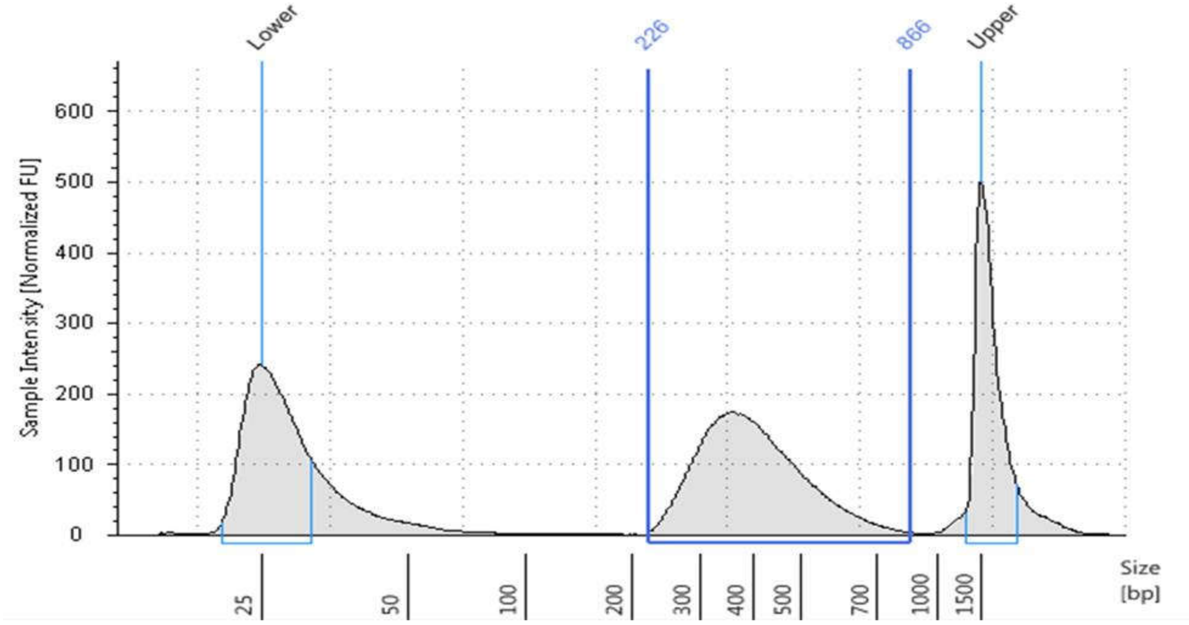

From [bp]	To [bp]	Average Size [bp]	Conc. [pg/µl]	Region Molarity [pm	% of Total
226	866	410	409	1640	74.91

Figure 3c: Tape Station profile of 198014

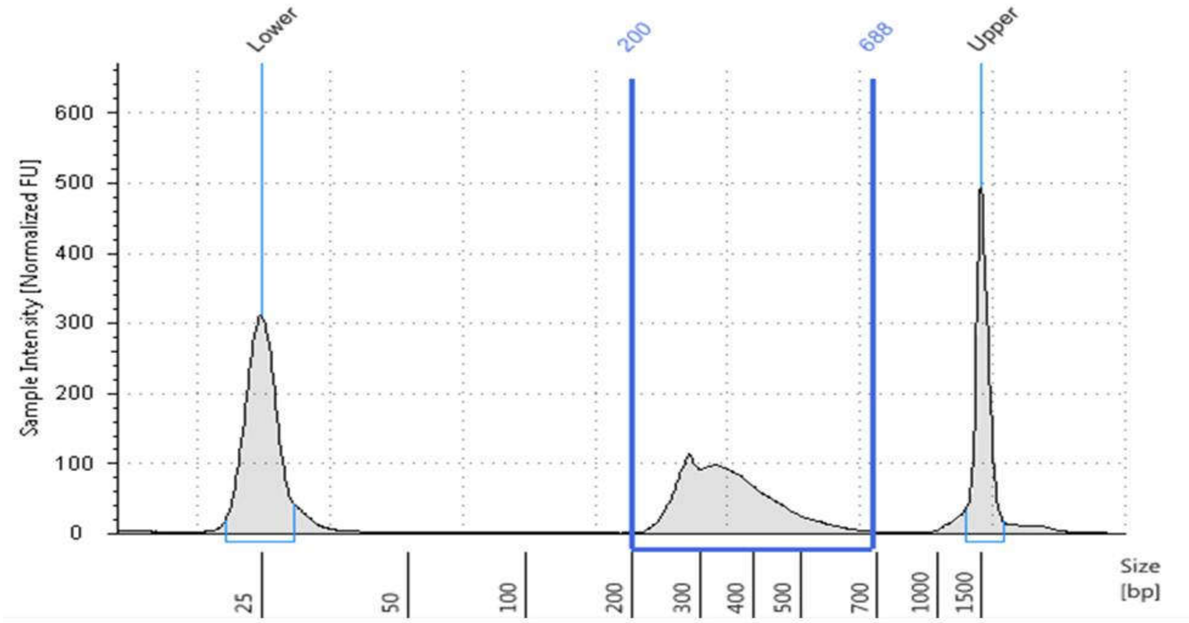

From [bp]	To [bp]	Average Size [bp]	Conc. [pg/µl]	Region Molarity [pm	% of Total
200	688	363	370	1660	82.40

Figure 3d: Tape Station profile of 198016

3.3 Primary Analysis

Raw Illumina reads were generated for 4 samples were in the range of 2.2-4.9 millions. The raw reads were trimmed for adapter sequence, quality (Q30) and length (20bp) using Trim galore to obtain good quality processed reads. The total numbers of raw and processed reads for all samples are given in table 3.

Table 3: Read statistics for 4 samples

Sample Name	Total Reads	Processed reads
1740786	2319869	2255902
1740787	4915287	4791452
198014	2950627	2888384
198016	2289644	2248574

3.4 Secondary Analysis

3.4.1 Host Contamination Removal

The processed Illumina reads from 4 samples were mapped against their respective host genomes using Bowtie2 tool. Two samples (1740786 & 1740787) were aligned to domestic pig genome (GCF_000003025.6) and other 2 samples (198014 & 198016) were aligned to water buffalo genome (GCF_003121395.1). After alignment against host genomes, the unmapped reads were filtered out by Samtools for downstream analysis.

Table 4: Read statistics for host unaligned reads

Sample Name	Processed reads	Unaligned Reads against Host
1740786	2255902	2227865
1740787	4791452	4783413
198014	2888384	2854450
198016	2248574	2230052

3.4.2 Reference Viral Genome based Alignment

The reads which are not aligned to host genome were filtered out and used further for mapping against specific reference rota virus genomes using Bowtie2. The 2 samples (1740786 & 1740787) were aligned to Porcine Rotavirus Gottfried G4P6 (GCA_002659895.1) and other 2 samples (198014 & 198016) were aligned to Bovine Rotavirus A strain NCDV G6. Fewer mapped reads obtained in case of samples (198014 & 198016) and hence excluded from further downstream analysis based on client's inputs.

3.4.3 Variant Prediction & annotation

The alignment data from 2 samples (1740786 & 1740787) were processed for variant calling & consensus generation approach using samtools pipeline. The predicted variants were used for annotation in snpEff tool. The total number of non-synonymous variants annotated in 2 samples was around 29-30. There is one non-synonymous SNP was annotated for VP7 gene in both the samples 1740786 and 1740787.

4. SUMMARY

Whole genome sequencing of 4 viral samples was carried out for performing variant analysis and annotation. Illumina sequencing of 4 samples generated raw data in the range of 2.2-4.9 million reads. The raw reads were processed based on phred quality score (Q30), length (minimum 20 bases) and good quality processed data was aligned against host genomes for removal of contaminants. The host specific unmapped reads were later used for mapping against reference viral genomes (Porcine Rotavirus Gottfried G4P6 & Bovine Rotavirus A strain NCDV G6) for variant prediction, consensus generation and annotation. Due to less number of reads (from samples 198014 & 198016) mapping against Bovine Rotavirus, both samples were excluded from further downstream analysis. The variants predicted for 2 samples (1740786 & 1740787) were around 29-30. The predicted variants were annotated in order to retrieve mutation effect at protein level and related useful information.

5. REFERENCES

1) https://support.illumina.com/sequencing/sequencing_software/bcl2fastq-conversion-software.html; RRID: SCR_015058

2) Andrews, S. FASTQC. A quality control tool for high throughput sequence data. (2010).

3) https://www.bioinformatics.babraham.ac.uk/projects/trim_galore/

4) Langmead B, Salzberg SL. Fast gapped-read alignment with Bowtie 2. Nat Methods. 2012;9(4):357-359. Published 2012 Mar 4. doi:10.1038/nmeth.1923

5) Li H, Handsaker B, Wysoker A, Fennell T, Ruan J, Homer N, Marth G, Abecasis G, Durbin R; 1000 Genome Project Data Processing Subgroup. The Sequence Alignment/Map format and SAMtools. Bioinformatics. 2009 Aug 15;25(16):2078-9. doi: 10.1093/bioinformatics/btp352. Epub 2009 Jun 8. PMID: 19505943; PMCID: PMC2723002.

6) "A program for annotating and predicting the effects of single nucleotide polymorphisms, SnpEff: SNPs in the genome of Drosophila melanogaster strain w1118; iso-2; iso-3.", Cingolani P, Platts A, Wang le L, Coon M, Nguyen T, Wang L, Land SJ, Lu X, Ruden DM. Fly (Austin). 2012 Apr-Jun;6(2):80-92. PMID: 22728672
