# Supplementary material for: Metatranscriptomic assessment of diarrhoeic faeces reveals diverse RNA viruses in rotavirus group A infected piglets and calves from India
Source: Front Cell Infect Microbiol. 2023 Oct 27;13:1258660. doi: 10.3389/fcimb.2023.1258660 (PMC10642067; doi:10.3389/fcimb.2023.1258660)
Supplement: Supplementary file 2 [file DataSheet_2.pdf]

Isolation of porcine RVA and confirmation by ELISA

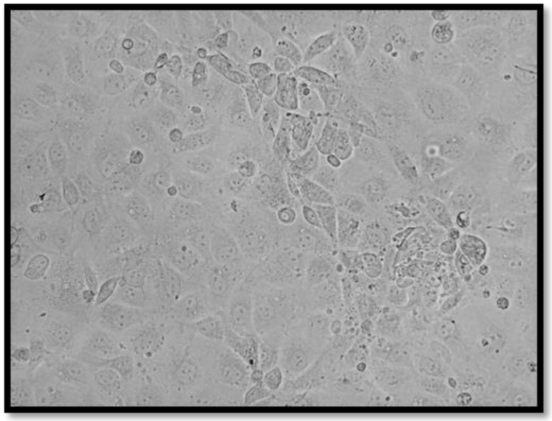

Figure: Cell control

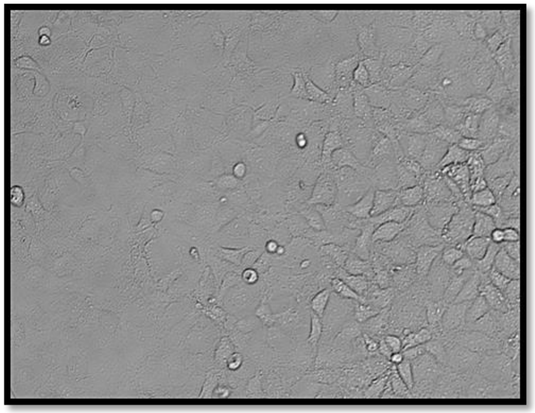

Figure: Sample no. NIV-1740786

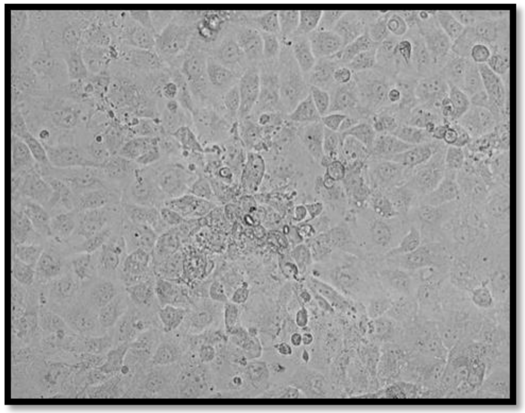

Figure: Sample No.: NIV-1740787

Figure: On 2nd day of the 4th passage of porcine samples: By comparing cell control, in sample, NIV-1740786 rounding and aggregation of the cells observed. In sample, NIV-1740787 cell aggregation and rounding of the cells has been increased.

Table: Ct values of Real-Time RT-PCR (NSP3) and OD values of ELISA (Passage 4) :

Sample description	Ct values		OD values	
	Sample No.	Ct Value	Sample No.	OD 450
Pig samples	1740786	30.96	1740786	1.770
Pig samples	1740787	30.84	1740787	1.512
Human RVA positive sample	Positive Control	24.28	Positive Control	1.667
RVA negative sample	Negative Control	NA	Negative Control	0.043

Detection of virus growth by NSP3 Real-Time PCR (Passage 4):

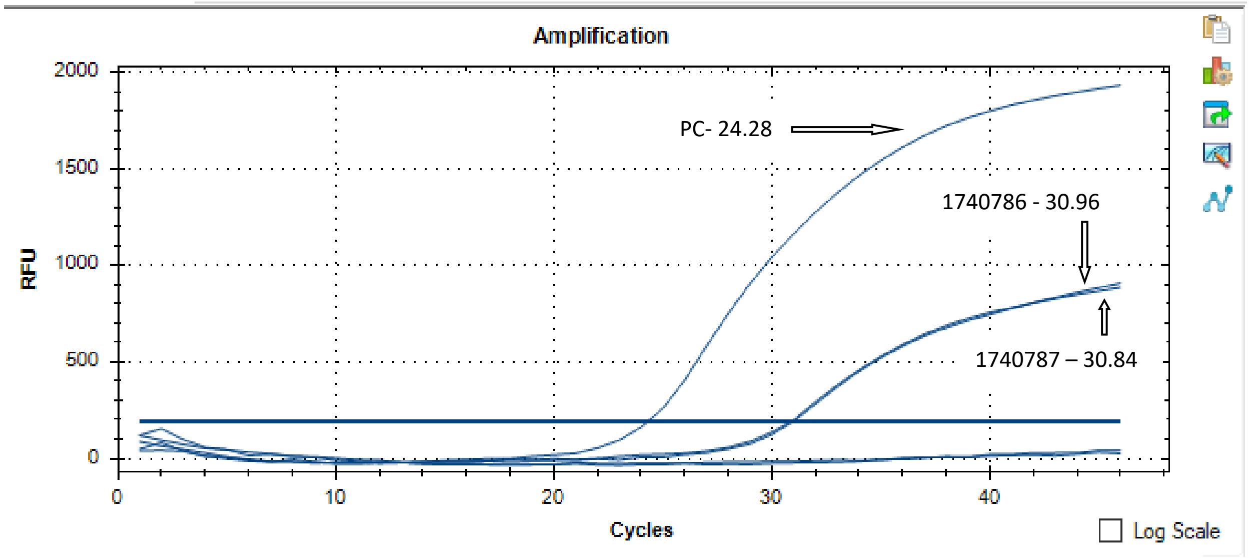

Figure: Amplification curves of the passage 4 samples

Table 1: Diversity of sequences identified in porcine faecal samples and their per cent identity and coverage from sample no. NIV1740786

qaccver	saccver	pident	length	mismatch	gapopen	qstart	qend	sstart	send	bitscore	evalue	qcovs
contig00001	KM977675.1	89.713	8049	809	18	4	8043	8076	38	10259	0	99
contig00002	KJ821021.1	87.778	7503	910	7	1	7499	25	7524	8769	0.00E+00	100
contig00003	MW145169.1	86.295	6939	934	17	107	7038	1	6929	7529	0.00E+00	98
contig00004	KX060809.1	84.711	6678	972	39	2	6656	6652	1	6630	0.00E+00	99
contig00005	OM104029.1	89.013	4396	468	12	2250	6636	4390	1	5428	0.00E+00	66
contig00006	MW784083.1	83.34	5204	830	30	7	5190	6	5192	4772	0.00E+00	86
contig00006	MW784083.1	77.083	336	60	10	6079	6406	6105	6431	178	2.80E-39	86
contig00007	MN734577.1	99.787	3286	2	3	1	3281	3303	18	6024	0.00E+00	99
contig00008	GU199192.1	97.157	2638	73	2	56	2692	2647	11	4455	0.00E+00	98
contig00009	MT876640.1	96.405	2587	93	0	1	2587	4	2590	4263	0.00E+00	100
contig00010	MW977860.1	79.087	2453	443	56	7	2422	2427	8	1624	0.00E+00	99
contig00011	KM393170.1	97.285	1989	54	0	1	1989	3284	1296	3374	0.00E+00	100
contig00012	KM393173.1	95.508	1536	69	0	1	1536	1	1536	2455	0.00E+00	100
contig00013	MN734577.1	99.497	1392	0	7	1	1390	7378	5992	2525	0.00E+00	100
contig00014	KM393170.1	97.424	1320	34	0	1	1320	1320	1	2250	0.00E+00	99
contig00015	KX363402.1	87.622	1131	140	0	7	1137	2352	1222	1314	0.00E+00	99
contig00016	MN066814.1	95.558	1103	49	0	1	1103	13	1115	1766	0.00E+00	99
contig00017	MN734577.1	98.639	1102	13	2	1	1101	4293	3193	1951	0.00E+00	100
contig00018	KF726074.1	94.934	1066	54	0	1	1066	10	1075	1670	0.00E+00	100
contig00019	M86490.1	97.019	1040	31	0	8	1047	1051	12	1749	0.00E+00	99
contig00020	KM393174.1	97.209	1039	29	0	1	1039	1053	15	1759	0.00E+00	100
contig00021	KX638599.1	97.443	1017	26	0	1	1017	1025	9	1735	0.00E+00	100
contig00022	MN734577.1	100	954	0	0	1	954	6102	5149	1762	0.00E+00	100
contig00023	MN734577.1	99.648	853	0	1	1	850	5147	4295	1555	0.00E+00	100
contig00024	AY601542.1	97.771	673	15	0	1	673	732	60	1160	0.00E+00	100
contig00025	MK105828.1	99.552	223	1	0	1	223	606	828	407	9.77E-110	100
contig00026	MN734577.1	99.552	223	1	0	1	223	6214	5992	407	9.77E-110	100
contig00027	MN734577.1	100	223	0	0	1	223	5037	5259	412	2.10E-111	100
contig00028	MN734577.1	99.552	223	1	0	1	223	4405	4183	407	9.77E-110	100
contig00029	MN734577.1	99.552	223	1	0	1	223	5037	5259	407	9.77E-110	100
contig00030	MN734577.1	100	223	0	0	1	223	4183	4405	412	2.10E-111	100

Table 2: Diversity of sequences identified in porcine faecal samples and their per cent identity and coverage from sample no. **NIV1740787**

qaccver	saccver	pident	length	mismatch	gapopen	qstart	qend	sstart	send	bitscore	evalue	qcovs
contig00001	MF782664.1	91.48	7570	633	10	1	7567	460	8020	10395	0	100
contig00002	KJ821021.1	87.724	7462	909	7	3	7460	44	7502	8698	0.00E+00	99
contig00003	MN734577.1	99.363	7375	30	13	4	7367	16	7384	13343	0.00E+00	99
contig00004	KX060809.1	84.711	6678	972	39	27	6681	6652	1	6630	0.00E+00	99
contig00005	MW784083.1	83.337	5203	830	30	1221	6403	5192	7	4771	0.00E+00	86
contig00005	MW784083.1	77.083	336	60	10	5	332	6431	6105	178	2.80E-39	86
contig00006	KT892971.1	87.992	4747	559	7	1	4741	5175	434	5598	0.00E+00	100
contig00007	OM104029.1	89.694	4124	410	12	6	4120	4118	1	5247	0.00E+00	99
contig00008	KM393170.1	97.411	3283	85	0	16	3298	1	3283	5592	0.00E+00	99
contig00009	GU199192.1	97.157	2638	73	2	7	2643	11	2647	4455	0.00E+00	98
contig00010	OP643775.1	86.06	2561	325	20	52	2601	1	2540	2723	0.00E+00	97
contig00011	MT876640.1	96.405	2587	93	0	1	2587	2588	2	4263	0.00E+00	100
contig00012	JX156399.2	87.591	2345	291	0	8	2352	2355	11	2719	0.00E+00	99
contig00013	KM393173.1	95.587	1541	68	0	2	1542	1	1541	2470	0.00E+00	99
contig00014	KX638599.1	97.615	1342	32	0	1	1342	10	1351	2302	0.00E+00	100
contig00015	KF726074.1	94.915	1062	54	0	7	1068	1067	6	1663	0.00E+00	96
contig00016	M86490.1	96.771	1053	33	1	2	1053	1061	9	1755	0.00E+00	99
contig00017	KM393174.1	97.19	1032	29	0	1	1032	1056	25	1746	0.00E+00	100
contig00018	AY601542.1	97.925	723	15	0	1	723	12	734	1253	0.00E+00	99
contig00019	AB741659.1	99.046	629	6	0	1	629	641	13	1129	0.00E+00	100

Table 3: Diversity of sequences identified in bovine faecal samples and their per cent identity and coverage from sample no. NIV198014

qaccver	saccver	pident	length	mismatch	gapopen	qstart	qend	sstart	send	bitscore	evalue	qcovs
contig00002	ON682281.1	76.769	4933	1082	58	38	4940	105	5003	2702	0	98
contig00003	ON682297.1	86.074	1429	199	0	2	1430	1429	1	1537	0	99
contig00005	MW373713.1	83.992	1037	166	0	1	1037	2356	1320	996	0	100
contig00006	ON624268.1	88.714	381	38	5	6	383	6308	5930	460	3.84E-125	37
contig00009	LC047797.1	90.732	615	57	0	2	616	3350	3964	821	0	96
contig00010	MW373714.1	87.402	635	80	0	1	635	2814	3448	730	0	100

contig00011	HQ916314.1	80.891	539	92	10	1	535	3925	4456	414	1.67E-111	93
contig00012	MK378521.1	84.874	119	18	0	93	211	4527	4645	121	3.83E-23	22
contig00015	ON624277.1	90.145	345	34	0	1	345	2922	2578	449	2.64E-122	99
contig00016	LC047797.1	91.589	321	27	0	1	321	2699	2379	444	1.15E-120	98
contig00018	ON682272.1	91.031	223	20	0	1	223	2536	2314	302	4.74E-78	100
contig00019	KY412126.1	79.803	203	41	0	15	217	543	745	148	6.54E-32	91
contig00020	HM756260.1	93.846	65	4	0	155	219	2704	2768	99	6.68E-17	29
contig00022	ON682272.1	90.498	221	21	0	1	221	2316	2536	292	2.82E-75	100
contig00023	OQ198051.1	91.364	220	19	0	2	221	3818	4037	302	4.69E-78	99
contig00026	OQ198051.1	91.364	220	18	1	2	220	3818	4037	300	1.68E-77	99
contig00031	ON624280.1	81.988	161	25	4	1	159	6087	5929	134	1.77E-27	73
contig00032	ON624281.1	78.704	108	21	2	4	110	1233	1127	71.3	0.000000014	50
contig00035	OQ198051.1	89	200	22	0	4	203	3030	2831	248	5.67E-62	98
contig00036	ON624281.1	82.692	156	27	0	48	203	1159	1004	139	3.56E-29	76
contig00038	ON624281.1	82.051	156	28	0	48	203	1159	1004	134	1.66E-27	76
contig00039	OQ198051.1	92.157	204	16	0	1	204	3836	4039	289	3.32E-74	100
contig00040	ON624277.1	88.542	192	22	0	1	192	2892	2701	233	1.48E-57	99
contig00041	KJ620979.1	86.458	192	26	0	1	192	2842	2651	211	6.92E-51	99
contig00043	KJ620979.1	86.979	192	25	0	1	192	2842	2651	217	1.49E-52	99
contig00045	ON624277.1	90.86	186	17	0	4	189	4050	3865	250	1.44E-62	98
contig00046	ON624277.1	91.398	186	16	0	4	189	4050	3865	255	3.09E-64	98
contig00054	ON624281.1	82.39	159	28	0	21	179	1159	1001	139	3.06E-29	88
contig00055	LC047797.1	92.135	178	14	0	1	178	3662	3485	252	3.72E-63	99
contig00056	ON010677.1	88.764	178	20	0	1	178	418	241	219	3.78E-53	99
contig00057	LC047797.1	91.573	178	15	0	1	178	3662	3485	246	1.73E-61	99
contig00058	ON624281.1	83.333	156	26	0	18	173	1159	1004	145	6.32E-31	89
contig00059	OQ198051.1	88.506	174	18	1	4	175	3030	2857	209	2.21E-50	98
contig00060	HQ916314.1	90.503	179	12	3	1	175	4038	3861	231	4.72E-57	100
contig00061	HQ916314.1	91.061	179	11	3	1	175	4038	3861	237	1.01E-58	100
contig00062	ON624281.1	82.051	156	28	0	3	158	1004	1159	134	1.37E-27	89
contig00063	ON624281.1	82.692	156	27	0	3	158	1004	1159	139	2.94E-29	89
contig00065	LC047797.1	91.176	170	15	0	1	170	3654	3485	231	4.58E-57	99
contig00066	LC047797.1	90.588	170	16	0	2	171	3485	3654	226	2.13E-55	99

contig00067	JF796127.1	85.965	171	24	0	1	171	207	377	183	1.3E-42	100
contig00068	ON624281.1	95.395	152	7	0	19	170	4027	3876	243	2.1E-60	89
contig00069	ON624281.1	96.053	152	6	0	19	170	4027	3876	248	4.52E-62	89
contig00082	OQ198051.1	89.809	157	16	0	4	160	3030	2874	202	3.44E-48	95
contig00085	JF796127.1	86.503	163	22	0	1	163	215	377	180	1.59E-41	100
contig00089	ON624277.1	91.72	157	13	0	1	157	3628	3472	219	3.23E-53	99
contig00090	ON624277.1	91.083	157	14	0	1	157	3628	3472	213	1.5E-51	99
contig00091	ON624277.1	91.613	155	13	0	1	155	3626	3472	215	4.11E-52	99
contig00092	HQ916314.1	88.722	133	10	3	28	156	4056	3925	158	7.03E-35	83
contig00093	ON624277.1	90.968	155	14	0	1	155	3626	3472	209	1.91E-50	99
contig00094	ON624281.1	80.132	151	28	2	2	151	1070	1219	111	5.55E-21	96
contig00095	ON624277.1	91.613	155	13	0	1	155	3626	3472	215	4.11E-52	99
contig00096	KJ476837.1	86.986	146	19	0	3	148	123	268	165	4.17E-37	94
contig00097	KJ476837.1	86.986	146	19	0	3	148	123	268	165	4.17E-37	94
contig00098	JF796127.1	87.742	155	19	0	1	155	223	377	182	4.14E-42	100
contig00099	ON624281.1	80.132	151	28	2	3	152	1219	1070	111	5.42E-21	98
contig00100	ON624281.1	80.132	151	28	2	3	152	1219	1070	111	5.42E-21	98
contig00109	ON624281.1	79.054	148	29	2	1	147	1114	1260	100	1.11E-17	100
contig00113	ON624281.1	78.767	146	29	2	1	145	1114	1258	97.1	1.42E-16	100
contig00114	ON624281.1	94.366	142	8	0	2	143	3970	3829	219	2.87E-53	99
contig00115	HQ916314.1	89.474	133	12	2	1	132	3925	4056	167	1.05E-37	92
contig00121	KJ620979.1	89.286	140	15	0	2	141	2871	2732	176	1.7E-40	99
contig00122	ON624281.1	87.778	90	11	0	2	91	1070	1159	106	2.21E-19	65
contig00123	ON624284.1	91.791	134	11	0	1	134	3672	3539	187	7.51E-44	99
contig00125	ON624281.1	86.667	90	12	0	45	134	1159	1070	100	9.97E-18	67
contig00126	JF796127.1	85.821	134	19	0	1	134	244	377	143	1.62E-30	100
contig00127	OP076765.1	85.455	110	16	0	4	113	3453	3562	115	3.45E-22	83
contig00128	ON624281.1	80.469	128	23	2	6	132	1219	1093	97.1	1.25E-16	96
contig00129	ON624281.1	80.469	128	23	2	1	127	1093	1219	97.1	1.25E-16	96
contig00131	ON624277.1	90.698	129	12	0	1	129	2798	2670	172	1.96E-39	100
contig00132	OQ198051.1	88.618	123	14	0	7	129	3045	2923	150	9.18E-33	95
contig00133	ON624281.1	79.688	128	24	2	1	127	1093	1219	91.6	5.65E-15	98
contig00137	HQ916314.1	95.122	123	6	0	3	125	3969	3847	195	4.05E-46	98

contig00138	HQ916314.1	94.309	123	7	0	2	124	3847	3969	189	1.89E-44	98
contig00139	ON624281.1	80.8	125	22	2	1	124	1096	1219	97.1	1.18E-16	98
contig00140	KJ476837.1	87.097	124	16	0	1	124	246	123	141	5.35E-30	98
contig00141	LC047797.1	91.803	122	10	0	3	124	3533	3654	171	6.76E-39	98
contig00142	KM822593.1	93.548	124	8	0	1	124	3642	3765	185	2.41E-43	100
contig00143	OQ198051.1	88.618	123	14	0	2	124	3045	2923	150	8.8E-33	99
contig00144	ON624284.1	91.057	123	11	0	2	124	3563	3685	167	8.74E-38	99
contig00145	ON624277.1	91.057	123	11	0	2	124	2792	2670	167	8.74E-38	99
contig00146	OQ198051.1	87.805	123	15	0	1	123	3063	2941	145	4.05E-31	100
contig00147	OQ198051.1	88.618	123	12	1	1	121	3043	2921	148	3.06E-32	100
contig00148	OQ198051.1	87.805	123	13	1	1	121	3043	2921	143	1.42E-30	100
contig00152	ON682272.1	88.393	112	13	0	8	119	3596	3485	135	2.33E-28	94
contig00153	LC047797.1	91.597	119	10	0	1	119	3544	3662	165	2.97E-37	100
contig00155	OQ198051.1	89.744	117	12	0	1	117	3043	2927	150	8.13E-33	100
contig00157	OQ198051.1	90.598	117	11	0	1	117	3946	3830	156	1.75E-34	100
contig00159	ON624284.1	90.991	111	10	0	1	111	3575	3685	150	8.13E-33	95
contig00160	ON624284.1	91.892	111	9	0	7	117	3685	3575	156	1.75E-34	95
contig00161	OQ198051.1	89.091	110	12	0	7	116	3045	2936	137	6.26E-29	95
contig00162	HQ916314.1	90.598	117	9	2	1	116	4035	3920	154	6.21E-34	100
contig00163	HQ916314.1	95.699	93	4	0	24	116	4012	3920	150	8.04E-33	80
contig00164	HQ916314.1	89.744	117	10	2	1	116	3920	4035	148	2.89E-32	100
contig00165	HQ916314.1	94.624	93	5	0	1	93	3920	4012	145	3.74E-31	80
contig00166	ON682272.1	90.909	110	10	0	7	116	2315	2424	148	2.89E-32	95
contig00167	OQ198051.1	91.346	104	9	0	5	108	2927	3030	143	1.33E-30	90
contig00168	ON624277.1	90.435	115	11	0	1	115	2670	2784	152	2.21E-33	100
contig00170	OQ198051.1	90.909	110	10	0	5	114	2927	3036	148	2.86E-32	96
contig00172	ON624281.1	87.097	93	12	0	2	94	1067	1159	106	1.72E-19	82
contig00173	ON624281.1	79.817	109	20	2	6	113	1219	1112	78.7	3.75E-11	95
contig00175	OQ198051.1	89.381	113	12	0	2	114	3045	2933	143	1.31E-30	99
contig00176	ON624281.1	80.734	109	19	2	2	109	1112	1219	84.2	8.07E-13	95
contig00178	ON682272.1	90.991	111	10	0	3	113	2316	2426	150	7.75E-33	98
contig00179	ON682272.1	90.826	109	10	0	5	113	2316	2424	147	1E-31	96
contig00180	KJ476833.1	91.964	112	9	0	1	112	410	299	158	4.63E-35	99

contig00182	KM822593.1	93.805	113	7	0	1	113	3640	3752	171	5.95E-39	100
contig00183	ON682272.1	90.826	109	10	0	3	111	2316	2424	147	1E-31	96
contig00184	ON682272.1	90.991	111	10	0	2	112	2315	2425	150	7.65E-33	99
contig00186	ON682272.1	90.909	110	10	0	1	110	2315	2424	148	2.75E-32	98
contig00187	OQ198051.1	91.346	104	9	0	4	107	3030	2927	143	1.28E-30	93
contig00188	ON682272.1	90.991	111	10	0	1	111	2314	2424	150	7.65E-33	99
contig00189	ON682272.1	90.909	110	10	0	2	111	2315	2424	148	2.75E-32	98

Table 4: Diversity of sequences identified in bovine faecal samples and their per cent identity and coverage from sample no. NIV198016

qaccver	saccver	pident	length	mismatch	gapopen	qstart	qend	sstart	send	bitscore	evalue	qcovs
contig00001	ON624260.1	80.556	2808	527	16	2	2800	1103	3900	2143	0	96
contig00002	ON624261.1	88.492	252	29	0	38	289	6048	5797	305	2.94E-78	16
contig00004	ON682281.1	76.608	171	38	2	1084	1253	316	147	93.5	2.11E-14	13
contig00013	MW373713.1	87.027	370	48	0	1	370	1987	2356	418	7.98E-113	100
contig00014	MW373714.1	87.535	353	44	0	3	355	3166	2814	409	4.59E-110	99
contig00015	MW504557.1	78.247	308	65	2	1	307	4478	4172	196	3.44E-46	97
contig00016	KJ476837.1	86.545	275	37	0	1	275	298	24	303	1.69E-78	99
contig00017	MK378521.1	85.057	87	12	1	1	87	4560	4645	87.9	1.75E-13	33
contig00018	KY412126.1	79.31	232	48	0	15	246	543	774	163	2.72E-36	91
contig00019	ON624280.1	85.714	112	14	2	32	142	5982	6092	117	2.12E-22	75
contig00019	ON624280.1	85.714	112	14	2	111	221	6092	5982	117	2.12E-22	75
contig00020	HQ916314.1	89.552	134	12	2	1	133	3925	4057	169	5.70E-38	100
contig00020	HQ916314.1	89.552	134	12	2	118	250	4057	3925	169	5.70E-38	100
contig00021	JQ696855.1	77.57	107	23	1	40	145	437	543	63.9	2.73E-06	68
contig00021	JQ696855.1	77.57	107	23	1	102	207	543	437	63.9	2.73E-06	68
contig00022	MW373713.1	82.203	236	42	0	3	238	1606	1371	204	1.48E-48	99
contig00023	HM756260.1	90.123	81	8	0	24	104	2704	2784	106	4.26E-19	69
contig00023	HM756260.1	90.123	81	8	0	133	213	2784	2704	106	4.26E-19	69
contig00024	ON624280.1	92.857	126	9	0	1	126	6122	6247	183	1.91E-42	100
contig00024	ON624280.1	92.857	126	9	0	111	236	6247	6122	183	1.91E-42	100
contig00025	JQ696855.1	83.168	101	17	0	11	111	437	537	93.5	3.19E-15	89
contig00025	JQ696855.1	83.168	101	17	0	118	218	537	437	93.5	3.19E-15	89

contig00026	ON624284.1	88.976	127	14	0	1	127	2667	2793	158	1.11E-34	56
contig00028	ON624281.1	79.556	225	44	2	1	224	1299	1076	159	3.04E-35	100
contig00029	OQ198051.1	92.793	222	16	0	1	222	3831	4052	322	3.62E-84	100
contig00030	MW373713.1	86.385	213	29	0	8	220	1466	1254	233	1.75E-57	96
contig00031	HQ916314.1	88.976	127	12	2	1	126	3931	4056	156	3.89E-34	57
contig00034	JQ696855.1	96.875	32	1	0	51	82	437	468	54.7	0.001	29
contig00034	JQ696855.1	96.875	32	1	0	141	172	468	437	54.7	0.001	29
contig00035	HQ916314.1	89.062	128	12	2	1	127	3931	4057	158	1.08E-34	57
contig00036	ON624280.1	92.063	126	10	0	96	221	6245	6120	178	8.25E-41	57
contig00038	MZ819164.1	91.071	56	5	0	159	214	4492	4547	76.8	3.08E-10	25
contig00040	ON624280.1	87.273	110	12	2	16	124	5982	6090	124	1.08E-24	86
contig00040	ON624280.1	87.273	110	12	2	97	205	6090	5982	124	1.08E-24	86
contig00041	ON682272.1	86.449	214	29	0	1	214	2165	1952	235	4.80E-58	97
contig00042	ON624280.1	85.981	107	15	0	16	122	5982	6088	115	6.53E-22	86
contig00042	ON624280.1	85.981	107	15	0	99	205	6088	5982	115	6.53E-22	86
contig00043	OQ198051.1	88.128	219	26	0	2	220	3737	3955	261	7.92E-66	99
contig00045	HM756260.1	88.298	94	9	2	16	108	2704	2796	111	8.44E-21	85
contig00045	HM756260.1	88.298	94	9	2	113	205	2796	2704	111	8.44E-21	85
contig00046	MK378521.1	90.625	64	6	0	1	64	4590	4527	86.1	5.09E-13	29
contig00048	LC047797.1	91.781	219	18	0	1	219	2695	2477	305	3.59E-79	100
contig00049	MW373713.1	88.732	213	24	0	7	219	3282	3070	261	7.88E-66	97
contig00052	MW373713.1	88.479	217	25	0	1	217	3272	3056	263	2.19E-66	99
contig00053	ON682272.1	90.868	219	20	0	1	219	2314	2532	294	7.77E-76	100
contig00054	ON624280.1	92.683	123	7	2	91	212	6198	6077	176	2.94E-40	97
contig00054	ON624280.1	94.595	111	6	0	1	111	6088	6198	172	3.80E-39	97
contig00055	HQ916314.1	90.863	197	13	3	27	219	4056	3861	259	2.83E-65	88
contig00057	MN150125.1	86.636	217	29	0	1	217	3191	3407	241	1.03E-59	99
contig00059	ON682272.1	90.411	219	21	0	1	219	2314	2532	289	3.61E-74	100
contig00063	LC047797.1	90.278	216	21	0	2	217	3485	3700	283	1.66E-72	99
contig00064	HQ916314.1	98.131	107	2	0	1	107	3894	4000	187	1.33E-43	99
contig00064	HQ916314.1	97.222	108	3	0	109	216	4008	3901	183	1.73E-42	99
contig00066	HQ916314.1	95.798	119	5	0	98	216	4012	3894	193	2.87E-45	100
contig00066	HQ916314.1	97.938	97	2	0	1	97	3901	3997	169	4.83E-38	100

contig00067	HQ916314.1	97.297	111	3	0	1	111	3894	4004	189	3.71E-44	100
contig00067	HQ916314.1	97.143	105	3	0	112	216	4005	3901	178	8.03E-41	100
contig00068	HQ916314.1	96.396	111	4	0	1	111	3894	4004	183	1.73E-42	100
contig00068	HQ916314.1	95.536	112	5	0	105	216	4012	3901	180	2.23E-41	100
contig00072	HQ916314.1	87.705	122	10	3	1	121	3940	4057	137	1.34E-28	57
contig00073	LC047797.1	90	210	21	0	4	213	3705	3496	272	3.52E-69	99
contig00075	MN150125.1	87.264	212	27	0	2	213	3214	3425	243	2.76E-60	99
contig00079	HQ916314.1	98.058	103	2	0	109	211	3997	3895	180	2.17E-41	100
contig00079	HQ916314.1	95.37	108	5	0	1	108	3905	4012	172	3.63E-39	100
contig00080	MW373713.1	89.005	191	21	0	1	191	24	214	237	1.27E-58	91
contig00081	MN231240.1	86.486	111	7	4	104	210	183	77	115	6.18E-22	100
contig00081	MN231240.1	84.426	122	8	5	1	118	70	184	110	2.87E-20	100
contig00083	ON624281.1	84.956	113	14	2	1	113	3965	4074	111	7.99E-21	100
contig00083	ON624281.1	83.898	118	16	2	93	210	4072	3958	110	2.87E-20	100
contig00084	MN231240.1	85.593	118	9	4	1	114	70	183	117	1.72E-22	100
contig00084	MN231240.1	83.621	116	8	5	99	210	185	77	99	6.22E-17	100
contig00086	ON624281.1	84.746	118	15	2	1	118	3958	4072	115	6.18E-22	100
contig00086	ON624281.1	84.685	111	14	2	100	210	4072	3965	108	1.03E-19	100
contig00088	MN231240.1	85.217	115	9	4	1	111	70	180	111	7.99E-21	100
contig00088	MN231240.1	83.621	116	8	5	99	210	185	77	99	6.22E-17	100
contig00089	ON624280.1	86.916	107	14	0	5	111	5982	6088	121	1.32E-23	91
contig00089	ON624280.1	86.111	108	13	2	88	194	6088	5982	115	6.14E-22	91
contig00090	ON624280.1	87.156	109	14	0	86	194	6090	5982	124	1.02E-24	91
contig00090	ON624280.1	86.364	110	13	2	5	113	5982	6090	119	4.75E-23	91
contig00091	ON624280.1	87.156	109	14	0	16	124	5982	6090	124	1.02E-24	91
contig00091	ON624280.1	86.111	108	13	2	99	205	6088	5982	115	6.14E-22	91
contig00092	ON624280.1	87.85	107	13	0	99	205	6088	5982	126	2.84E-25	91
contig00092	ON624280.1	85.185	108	14	2	16	122	5982	6088	110	2.86E-20	91
contig00093	ON682272.1	87.081	209	27	0	1	209	2165	1957	237	1.26E-58	100
contig00096	ON624280.1	87.273	110	12	2	10	118	5982	6090	124	1.01E-24	91
contig00096	ON624280.1	87.273	110	12	2	91	199	6090	5982	124	1.01E-24	91
contig00097	ON624280.1	85.981	107	15	0	10	116	5982	6088	115	6.11E-22	91
contig00097	ON624280.1	85.981	107	15	0	93	199	6088	5982	115	6.11E-22	91

contig00098	ON624280.1	87.037	108	12	2	10	116	5982	6088	121	1.31E-23	91
contig00098	ON624280.1	87.037	108	12	2	93	199	6088	5982	121	1.31E-23	91
contig00099	ON624280.1	88.393	112	11	2	3	113	5982	6092	134	1.68E-27	92
contig00099	ON624280.1	85.185	108	14	2	86	192	6088	5982	110	2.83E-20	92
contig00100	ON624280.1	87.387	111	14	0	3	113	5982	6092	128	7.80E-26	92
contig00100	ON624280.1	85.185	108	14	2	86	192	6088	5982	110	2.83E-20	92
contig00101	ON624280.1	87.156	109	14	0	97	205	6090	5982	124	1.01E-24	92
contig00101	ON624280.1	86.111	108	13	2	16	122	5982	6088	115	6.07E-22	92
contig00102	MN231240.1	84.211	114	10	4	1	110	74	183	104	1.31E-18	100
contig00102	MN231240.1	84.211	114	10	4	97	206	183	74	104	1.31E-18	100
contig00104	MN231240.1	86.486	111	7	4	2	108	77	183	115	6.00E-22	99
contig00104	MN231240.1	84.483	116	10	4	94	205	184	73	108	1.00E-19	99
contig00105	OQ198051.1	92.195	205	16	0	1	205	3848	4052	291	9.29E-75	100
contig00107	ON624280.1	87.963	108	11	2	5	111	5982	6088	126	2.74E-25	94
contig00107	ON624280.1	86.111	108	13	2	88	194	6088	5982	115	5.93E-22	94
contig00108	MN231240.1	86.111	108	7	4	100	203	180	77	110	2.76E-20	100
contig00108	MN231240.1	84.762	105	8	4	1	101	77	177	99	5.97E-17	100
contig00111	MN231240.1	86.364	110	7	4	1	106	78	183	113	2.12E-21	100
contig00111	MN231240.1	83.478	115	8	5	92	202	184	77	97.1	2.14E-16	100
contig00112	ON624280.1	88.182	110	11	2	91	199	6090	5982	130	2.09E-26	95
contig00112	ON624280.1	86.111	108	13	2	10	116	5982	6088	115	5.86E-22	95
contig00113	ON624280.1	88.073	109	13	0	91	199	6090	5982	130	2.09E-26	95
contig00113	ON624280.1	85.185	108	14	2	10	116	5982	6088	110	2.73E-20	95
contig00114	OQ198051.1	85.075	201	28	2	1	200	3145	2946	204	1.21E-48	100
contig00115	MW373713.1	87.179	195	25	0	6	200	3330	3136	222	3.34E-54	98
contig00116	MW373713.1	84.848	198	30	0	1	198	3352	3155	200	1.55E-47	100
contig00118	ON624280.1	81.481	135	21	4	1	133	6061	5929	108	9.64E-20	67
contig00121	ON624280.1	87.037	108	12	2	5	111	5982	6088	121	1.24E-23	96
contig00121	ON624280.1	87.037	108	12	2	88	194	6088	5982	121	1.24E-23	96
contig00122	MK378521.1	87.342	79	9	1	106	184	4527	4604	89.8	3.47E-14	40
contig00125	OP413962.1	80.921	152	21	6	47	195	1471	1617	113	2.03E-21	76
contig00126	ON624277.1	91.237	194	17	0	1	194	2803	2610	265	5.27E-67	100
contig00127	ON624277.1	88.542	192	22	0	1	192	2892	2701	233	1.48E-57	99

contig00129	KJ620979.1	86.979	192	25	0	2	193	2651	2842	217	1.49E-52	99
contig00134	MW373714.1	92.188	192	15	0	1	192	2527	2336	272	3.11E-69	100
contig00135	ON624280.1	88.288	111	13	0	1	111	5982	6092	134	1.53E-27	99
contig00135	ON624280.1	90	80	8	0	111	190	6061	5982	104	1.20E-18	99
contig00136	MZ603733.1	87.958	191	23	0	1	191	375	185	226	2.44E-55	100
contig00137	MW373713.1	89.529	191	20	0	1	191	433	243	243	2.42E-60	100
contig00138	ON682272.1	96.237	186	7	0	6	191	4002	3817	305	3.05E-79	97
contig00142	ON624281.1	79.57	186	36	2	2	186	1070	1254	132	5.34E-27	99
contig00143	HQ916314.1	92.222	180	12	2	1	179	4056	3878	254	1.09E-63	96
contig00145	HQ916314.1	92	175	12	2	1	174	3883	4056	244	6.53E-61	94
contig00146	KJ620979.1	86.486	185	25	0	1	185	2835	2651	204	1.11E-48	99
contig00148	ON624281.1	80.11	181	34	2	1	180	1093	1272	134	1.47E-27	97
contig00153	ON624268.1	93.491	169	10	1	1	168	6140	6308	250	1.38E-62	91
contig00159	ON624280.1	91.758	182	15	0	1	182	6180	5999	254	1.06E-63	100
contig00161	MK378521.1	84.821	112	17	0	69	180	4645	4534	113	1.85E-21	62
contig00162	MW373713.1	86.932	176	23	0	1	176	3155	3330	198	4.95E-47	98
contig00163	HQ916314.1	84.27	178	23	4	4	180	4098	3925	169	3.88E-38	98
contig00164	ON624281.1	93.258	178	12	0	1	178	4012	3835	263	1.71E-66	100
contig00166	OQ198051.1	89.08	174	19	0	4	177	3030	2857	217	1.34E-52	98
contig00167	ON624281.1	82.692	156	27	0	3	158	1004	1159	139	2.94E-29	89
contig00173	MW373713.1	84.049	163	26	0	1	163	1317	1479	158	8.00E-35	94
contig00175	ON682303.1	90.854	164	15	0	7	170	3236	3073	220	9.92E-54	96
contig00176	ON624277.1	88.889	171	19	0	1	171	2722	2892	211	5.97E-51	100
contig00177	MK378521.1	90.411	73	7	0	98	170	4527	4599	97.1	1.73E-16	43
contig00178	ON624277.1	91.176	170	15	0	1	170	2610	2779	231	4.55E-57	100
contig00180	MW373714.1	94.048	168	10	0	1	168	2573	2406	255	2.66E-64	100
contig00183	MW373714.1	93.75	160	10	0	1	160	2527	2368	241	7.34E-60	96
contig00187	ON624277.1	89.634	164	17	0	1	164	2885	2722	209	2.04E-50	100
contig00188	ON624269.1	88.272	162	17	2	4	164	2726	2566	193	2.05E-45	98
contig00190	MW373713.1	85.806	155	22	0	9	163	1466	1312	165	4.44E-37	95
contig00191	LC047797.1	92.025	163	13	0	1	163	3657	3495	230	1.55E-56	100
contig00197	HQ916314.1	95.455	88	4	0	74	161	4012	3925	141	7.37E-30	100
contig00197	HQ916314.1	97.26	73	2	0	1	73	3925	3997	124	7.42E-25	100

contig00198	ON624281.1	78.261	161	33	2	1	160	1131	1290	102	3.48E-18	99
contig00204	ON624280.1	82.222	135	20	4	1	133	6061	5929	113	1.56E-21	85
contig00210	ON624281.1	80.132	151	28	2	2	151	1070	1219	111	5.55E-21	96
contig00214	MW373713.1	88.961	154	17	0	1	154	476	323	191	6.82E-45	100
contig00215	MZ819164.1	91.071	56	5	0	93	148	4492	4547	76.8	1.99E-10	36
contig00216	ON624266.1	93.878	147	9	0	7	153	3821	3675	222	2.42E-54	95
contig00218	HQ916315.1	87.075	147	19	0	5	151	1840	1694	167	1.14E-37	96
contig00219	OP413962.1	81.457	151	21	6	1	148	1617	1471	117	1.16E-22	97
contig00220	ON682272.1	95.238	147	7	0	1	147	3856	4002	233	1.10E-57	97
contig00223	MN150125.1	86	150	21	0	1	150	3405	3256	161	5.17E-36	100
contig00224	ON624281.1	83.333	132	22	0	2	133	1028	1159	122	2.44E-24	88
contig00225	ON682298.1	90.728	151	13	1	1	150	2682	2532	200	1.10E-47	100
contig00226	ON624281.1	82.609	138	24	0	1	138	1022	1159	122	2.44E-24	92
contig00227	LC047797.1	90.604	149	14	0	1	149	3552	3700	198	3.91E-47	100
contig00228	LC047797.1	91.034	145	13	0	5	149	3485	3629	196	1.41E-46	97
contig00231	HM756260.1	100	36	0	0	3	38	2739	2704	67.6	1.15E-07	24
contig00232	HQ916314.1	97.297	111	3	0	39	149	3997	3887	189	2.35E-44	74
contig00234	MW373713.1	83.893	149	24	0	1	149	1332	1480	143	1.86E-30	100
contig00235	ON624277.1	91.781	146	12	0	1	146	2720	2575	204	8.33E-49	99
contig00236	HQ916314.1	93.197	147	10	0	1	147	3993	3847	217	1.07E-52	99
contig00237	HQ916314.1	93.878	147	9	0	1	147	3993	3847	222	2.30E-54	99
contig00240	HQ916314.1	93.878	147	9	0	1	147	3993	3847	222	2.30E-54	99
contig00242	MW373712.1	85.915	71	9	1	4	73	3967	4037	75	6.75E-10	48
contig00243	ON624281.1	82.609	138	24	0	1	138	1022	1159	122	2.38E-24	94
contig00245	ON624281.1	79.021	143	28	2	1	142	1131	1272	97.1	1.44E-16	97
contig00246	LC047797.1	90.58	138	13	0	1	138	3520	3657	183	1.08E-42	94
contig00247	MW373712.1	85.915	71	9	1	75	144	4037	3967	75	6.75E-10	48
contig00248	MK378521.1	84.034	119	19	0	24	142	4645	4527	115	3.98E-22	81
contig00255	MN231240.1	86.087	115	8	4	1	111	74	184	117	1.10E-22	76
contig00256	MN150125.1	90.654	107	10	0	37	143	3425	3319	143	1.81E-30	73
contig00257	MW373714.1	96.503	143	5	0	1	143	2573	2431	237	8.00E-59	99
contig00258	MW373712.1	94.737	38	1	1	109	145	4037	4000	58.4	6.68E-05	26
contig00262	ON624281.1	83.465	127	21	0	1	127	1033	1159	119	2.99E-23	88

contig00265	HQ916314.1	89.474	133	12	2	13	144	4056	3925	167	1.05E-37	92
contig00266	HQ916314.1	90.972	144	11	2	1	143	3893	4035	193	1.72E-45	100
contig00267	HQ916314.1	95.455	88	4	0	56	143	4012	3925	141	6.33E-30	62
contig00270	ON624280.1	93.706	143	9	0	1	143	6088	6230	215	3.68E-52	100
contig00274	JQ696855.1	80.556	72	14	0	1	72	508	437	56.5	2.34E-04	51
contig00275	ON682272.1	93.75	144	7	1	1	142	3779	3922	215	3.65E-52	100
contig00279	MW373713.1	90.78	141	13	0	2	142	3282	3142	189	2.21E-44	99
contig00290	KJ620979.1	89.286	140	15	0	2	141	2871	2732	176	1.70E-40	99
contig00297	ON682272.1	89.855	138	12	1	1	136	2759	2896	176	1.69E-40	97
contig00299	ON624277.1	88.571	140	16	0	1	140	2832	2693	171	7.86E-39	100
contig00301	ON682272.1	89.13	138	13	1	5	140	2896	2759	171	7.86E-39	97
contig00305	HQ916314.1	92.029	138	9	2	1	137	3902	4038	193	1.66E-45	99
contig00313	MW373713.1	89.855	138	14	0	1	138	3245	3108	178	4.61E-41	100
contig00314	LC047797.1	91.304	138	12	0	1	138	3520	3657	189	2.13E-44	100
contig00315	ON682272.1	91.176	136	12	0	3	138	2885	2750	185	2.75E-43	99
contig00316	ON624281.1	86.022	93	13	0	2	94	1067	1159	100	1.03E-17	67
contig00317	ON682293.1	82.653	98	17	0	40	137	3407	3310	87.9	7.91E-14	72
contig00318	MK378521.1	84.746	118	18	0	20	137	4527	4644	119	2.81E-23	86
contig00320	JQ696855.1	82.653	98	17	0	1	98	534	437	87.9	7.91E-14	72
contig00321	MK378521.1	84.746	118	18	0	1	118	4644	4527	119	2.81E-23	86
contig00323	HQ916314.1	95.455	88	4	0	1	88	3925	4012	141	5.99E-30	64
contig00325	OQ198051.1	95.522	134	6	0	3	136	3944	4077	215	3.48E-52	98
contig00328	BK046622.1	97.222	36	1	0	1	36	1	36	62.1	4.80E-06	26
contig00329	ON624281.1	94.118	136	8	0	2	137	3970	3835	207	5.82E-50	99
contig00330	MK378521.1	83.898	118	19	0	20	137	4527	4644	113	1.31E-21	86
contig00331	MK378521.1	83.898	118	19	0	20	137	4527	4644	113	1.31E-21	86
contig00336	MW373713.1	90.441	136	13	0	1	136	3269	3134	180	1.26E-41	100
contig00339	ON624280.1	87.619	105	13	0	1	105	6086	5982	122	2.15E-24	77
contig00341	ON682293.1	81.618	136	25	0	1	136	3445	3310	113	1.29E-21	100
contig00345	MW373712.1	94.737	38	1	1	1	37	4000	4037	58.4	6.14E-05	54
contig00345	MW373712.1	94.737	38	1	1	100	136	4037	4000	58.4	6.14E-05	54
contig00346	ON682293.1	82.353	136	24	0	1	136	3445	3310	119	2.78E-23	100
contig00351	MW504550.1	80.882	136	24	2	1	135	6064	6198	106	2.14E-19	100

contig00354	MK378521.1	84.821	112	17	0	1	112	4534	4645	113	1.28E-21	83
contig00356	MK378521.1	88.608	79	8	1	44	122	4527	4604	95.3	4.64E-16	59
contig00357	HQ916314.1	97.938	97	2	0	39	135	3997	3901	169	2.69E-38	72
contig00358	LC047797.1	91.791	134	11	0	1	134	3496	3629	187	7.36E-44	100
contig00361	MZ819164.1	91.071	56	5	0	33	88	4547	4492	76.8	1.66E-10	42
contig00366	KJ620979.1	88.55	131	15	0	4	134	2862	2732	159	1.61E-35	98
contig00368	MW373713.1	90.226	133	13	0	1	133	3108	3240	174	5.68E-40	100
contig00369	ON682297.1	92.248	129	10	0	5	133	1	129	183	9.43E-43	97
contig00374	ON682272.1	93.985	133	8	0	1	133	3790	3922	202	2.60E-48	100
contig00375	ON624281.1	79.688	128	24	2	6	132	1219	1093	91.6	5.82E-15	96
contig00382	HQ916314.1	90.909	132	10	2	1	131	4035	3905	176	1.55E-40	100
contig00384	HQ916314.1	95.37	108	5	0	24	131	4012	3905	172	2.00E-39	82
contig00386	HQ916314.1	96.296	108	4	0	24	131	4012	3905	178	4.30E-41	82
contig00387	JQ696855.1	96.875	32	1	0	30	61	468	437	54.7	7.56E-04	24
contig00395	MZ603733.1	89.231	130	14	0	1	130	407	278	163	1.19E-36	100
contig00396	MW373713.1	90.625	128	12	0	3	130	151	24	171	7.12E-39	98
contig00398	MZ819163.1	88	75	8	1	55	129	1285	1212	87.9	7.38E-14	58
contig00399	ON682292.1	90	130	13	0	1	130	354	225	169	2.56E-38	100
contig00401	ON682292.1	90	130	13	0	1	130	354	225	169	2.56E-38	100
contig00402	MZ603733.1	89.231	130	14	0	1	130	407	278	163	1.19E-36	100
contig00405	MN150125.1	88.976	127	14	0	3	129	3407	3281	158	5.49E-35	98
contig00406	ON682293.1	81.395	129	24	0	1	129	3292	3420	106	2.02E-19	100
contig00410	ON682293.1	80.62	129	25	0	1	129	3292	3420	100	9.38E-18	100
contig00412	ON624280.1	95.082	122	6	0	8	129	6077	6198	193	1.50E-45	95
contig00418	MW373713.1	90.099	101	10	0	29	129	3282	3182	132	3.33E-27	78
contig00420	ON624281.1	78.512	121	24	2	1	120	1114	1233	78.7	4.35E-11	94
contig00421	KJ620979.1	90	110	11	0	19	128	2841	2732	143	1.52E-30	86
contig00424	MW373713.1	86.777	121	16	0	8	128	1466	1346	135	2.54E-28	95
contig00425	ON624280.1	93.701	127	8	0	2	128	6248	6122	191	5.36E-45	99
contig00428	OQ198051.1	90.741	108	10	0	1	108	3842	3949	145	4.18E-31	85
contig00430	MK378521.1	85.057	87	12	1	1	87	4560	4645	87.9	7.15E-14	69
contig00431	MN150125.1	88.976	127	14	0	1	127	3425	3299	158	5.37E-35	100
contig00434	MW373714.1	96.8	125	4	0	3	127	2431	2555	209	1.46E-50	98

contig00435	MW373714.1	88.189	127	15	0	1	127	3184	3058	152	2.50E-33	100
contig00438	HQ916314.1	88.189	127	13	2	1	126	3931	4056	150	8.90E-33	100
contig00439	MW373713.1	86.179	123	17	0	1	123	366	488	134	8.96E-28	98
contig00441	MW373713.1	91.667	120	10	0	7	126	3249	3130	167	8.83E-38	95
contig00442	MW373713.1	91.667	120	10	0	7	126	3249	3130	167	8.83E-38	95
contig00447	HQ916314.1	95.122	123	6	0	3	125	3969	3847	195	4.05E-46	98
contig00454	MW373713.1	86.992	123	16	0	4	126	488	366	139	1.93E-29	98
contig00462	OQ198051.1	89.908	109	11	0	18	126	3956	3848	141	5.35E-30	87
contig00463	MW373713.1	92.5	120	9	0	7	126	3249	3130	172	1.90E-39	95
contig00467	ON682297.1	91.2	125	11	0	1	125	129	5	171	6.76E-39	100
contig00468	MW373713.1	90.323	124	12	0	2	125	3282	3159	163	1.13E-36	99
contig00473	MW373713.1	89.516	124	13	0	2	125	3282	3159	158	5.26E-35	99
contig00474	MW373714.1	95.37	108	5	0	18	125	2573	2466	172	1.88E-39	86
contig00475	MZ603733.1	92.727	110	8	0	1	110	266	375	159	1.46E-35	88
contig00477	MN231240.1	86.408	103	6	4	27	125	183	85	106	1.93E-19	79
contig00479	MW373713.1	90.323	124	12	0	2	125	3282	3159	163	1.13E-36	99
contig00483	MN231240.1	86.207	87	4	5	2	84	101	183	87.9	7.00E-14	66
contig00484	HM756260.1	90.123	81	8	0	16	96	2704	2784	106	1.93E-19	65
contig00485	OQ198051.1	88.618	123	14	0	2	124	3045	2923	150	8.80E-33	99
contig00486	MZ603733.1	93.043	115	8	0	10	124	379	265	169	2.43E-38	93
contig00487	ON624277.1	91.87	123	10	0	2	124	2792	2670	172	1.88E-39	99
contig00492	ON624277.1	91.057	123	11	0	2	124	2792	2670	167	8.74E-38	99
contig00493	MW373713.1	84.211	114	17	1	1	113	1371	1484	110	1.49E-20	91
contig00500	HQ916314.1	98.276	116	2	0	9	124	3878	3993	204	6.66E-49	94
contig00502	ON624277.1	91.057	123	11	0	2	124	2792	2670	167	8.74E-38	99
contig00503	MW373713.1	88.889	117	13	0	8	124	3289	3173	145	4.10E-31	94
contig00505	MW373714.1	95.935	123	5	0	1	123	2539	2417	200	8.52E-48	100
contig00508	MW373713.1	91.589	107	9	0	1	107	3166	3272	148	3.13E-32	87
contig00511	MW373714.1	95.935	123	5	0	1	123	2539	2417	200	8.52E-48	100
contig00513	HQ916314.1	95.833	120	5	0	4	123	4012	3893	195	3.97E-46	98
contig00514	ON682292.1	90.323	124	11	1	1	123	244	367	161	4.02E-36	100
contig00516	OQ198051.1	89.815	108	11	0	16	123	3955	3848	139	1.88E-29	88
contig00522	JF796127.1	86.885	122	16	0	2	123	268	389	137	6.78E-29	99

contig00526	KJ620979.1	90.385	104	10	0	20	123	2835	2732	137	6.78E-29	85
contig00527	MW373714.1	95.122	123	6	0	1	123	2539	2417	195	3.97E-46	100
contig00528	MW373714.1	96.748	123	4	0	1	123	2539	2417	206	1.83E-49	100
contig00535	ON682293.1	82.407	108	19	0	1	108	3310	3417	95.3	4.09E-16	89
contig00537	ON682272.1	91.597	119	10	0	3	121	2885	2767	165	3.08E-37	98
contig00538	HQ916314.1	96.296	108	4	0	15	122	4012	3905	178	3.95E-41	89
contig00541	ON682293.1	82.692	104	18	0	19	122	3413	3310	93.5	1.47E-15	85
contig00543	HQ916314.1	95.37	108	5	0	1	108	3905	4012	172	1.84E-39	89
contig00545	HQ916314.1	95.798	119	5	0	1	119	3894	4012	193	1.41E-45	98
contig00546	ON624280.1	88.43	121	14	0	1	121	6102	5982	147	1.10E-31	100
contig00547	HQ916314.1	88.136	118	12	2	1	117	3940	4056	139	1.84E-29	97
contig00548	ON682293.1	81.308	107	20	0	14	120	3407	3301	87.9	6.77E-14	88
contig00557	HQ916314.1	95.455	88	4	0	34	121	4012	3925	141	5.12E-30	73
contig00561	LC047797.1	93.396	106	7	0	1	106	3552	3657	158	5.09E-35	88
contig00562	HQ916314.1	86.885	122	11	3	1	121	3940	4057	132	3.08E-27	100
contig00565	OQ198051.1	88.43	121	14	0	1	121	3056	2936	147	1.10E-31	100
contig00567	MK378521.1	90.625	64	6	0	15	78	4590	4527	86.1	2.44E-13	53
contig00568	OP413962.1	86.022	93	11	2	9	100	1619	1528	99	3.13E-17	76
contig00569	MW373713.1	90.909	121	11	0	1	121	3265	3145	163	1.09E-36	100
contig00572	MW373713.1	90.909	121	11	0	1	121	3145	3265	163	1.09E-36	100
contig00573	ON682293.1	82.243	107	19	0	14	120	3407	3301	93.5	1.46E-15	88
contig00581	ON682293.1	82.692	104	18	0	1	104	3310	3413	93.5	1.44E-15	87
contig00587	MN231240.1	86.207	87	4	5	37	119	183	101	87.9	6.70E-14	69
contig00588	HQ916314.1	95.833	120	5	0	1	120	3980	3861	195	3.83E-46	100
contig00590	MW373713.1	92.982	114	8	0	7	120	3249	3136	167	8.36E-38	95
contig00591	HM447046.1	96.364	110	4	0	11	120	590	481	182	2.99E-42	92
contig00594	HM447046.1	96.04	101	4	0	1	101	490	590	165	3.01E-37	84
contig00596	HM447046.1	95.455	110	5	0	11	120	590	481	176	1.39E-40	92
contig00597	HM447046.1	94.167	120	7	0	1	120	481	600	183	8.30E-43	100
contig00603	HM447046.1	93.333	120	8	0	1	120	481	600	178	3.86E-41	100
contig00606	HQ916314.1	94.845	97	5	0	24	120	4012	3916	152	2.34E-33	81
contig00633	HQ916314.1	95.876	97	4	0	24	120	4012	3916	158	5.03E-35	81
contig00634	HM756260.1	90.123	81	8	0	16	96	2784	2704	106	1.85E-19	68

contig00638	ON682272.1	93.277	119	8	0	1	119	2751	2869	176	1.37E-40	100
contig00641	MZ603733.1	93.636	110	7	0	10	119	379	270	165	2.97E-37	92
contig00642	KJ620979.1	90.385	104	10	0	16	119	2835	2732	137	6.48E-29	87
contig00644	HM756260.1	100	34	0	0	86	119	2704	2737	63.9	1.12E-06	29
contig00647	HM756260.1	100	34	0	0	1	34	2737	2704	63.9	1.12E-06	29
contig00648	HM756260.1	88.298	94	9	2	24	116	2704	2796	111	3.93E-21	78
contig00649	ON624277.1	92.079	101	8	0	19	119	2819	2719	143	1.39E-30	85
contig00653	ON682293.1	81.897	116	21	0	1	116	3292	3407	99	3.06E-17	97
contig00657	ON624277.1	89.916	119	12	0	1	119	2829	2711	154	6.43E-34	100
contig00661	ON624277.1	91.743	109	9	0	11	119	2819	2711	152	2.31E-33	92
contig00664	ON682293.1	81.034	116	22	0	4	119	3407	3292	93.5	1.42E-15	97
contig00665	ON624277.1	90.826	109	10	0	11	119	2819	2711	147	1.08E-31	92
contig00667	ON624277.1	89.076	119	13	0	1	119	2829	2711	148	2.99E-32	100
contig00671	MW373713.1	84.483	116	18	0	1	116	1480	1365	115	3.00E-22	98
contig00675	KJ620979.1	90	110	11	0	8	117	2835	2726	143	1.38E-30	93
contig00676	HQ916314.1	90	110	9	2	1	109	3930	4038	141	4.95E-30	92
contig00678	ON624280.1	89.831	118	12	0	1	118	6109	5992	152	2.29E-33	100
contig00682	ON624284.1	91.509	106	9	0	1	106	3581	3686	147	1.06E-31	90
contig00687	OQ198051.1	87.288	118	15	0	1	118	3063	2946	135	2.30E-28	100
contig00691	ON624280.1	88.983	118	13	0	1	118	5992	6109	147	1.06E-31	100
contig00694	KJ620979.1	90.909	110	10	0	8	117	2835	2726	148	2.96E-32	93
contig00698	MW373714.1	94.915	118	6	0	1	118	3833	3950	185	2.26E-43	100
contig00700	HM447045.1	95.726	117	5	0	1	117	714	598	189	1.74E-44	99
contig00707	MW373714.1	94.068	118	7	0	1	118	3833	3950	180	1.05E-41	100
contig00711	MN231240.1	83.607	122	9	5	1	118	184	70	104	6.50E-19	100
contig00717	MN231240.1	86.364	110	7	4	13	118	183	78	113	1.08E-21	90
contig00719	MN231240.1	86.087	115	8	4	8	118	180	70	117	8.35E-23	94
contig00720	HM447045.1	94.872	117	6	0	2	118	598	714	183	8.11E-43	99
contig00723	ON682272.1	92.373	118	9	0	1	118	2759	2876	169	2.27E-38	100
contig00726	MW373713.1	91.453	117	10	0	2	118	3282	3166	161	3.80E-36	99
contig00727	ON682293.1	82.569	109	19	0	10	118	3418	3310	97.1	1.09E-16	92
contig00730	HQ916314.1	98.058	103	2	0	1	103	3895	3997	180	1.04E-41	88
contig00735	ON624280.1	84.211	114	16	2	5	117	6070	5958	110	1.38E-20	97

contig00736	ON624280.1	86.408	103	12	2	16	117	5982	6083	111	3.84E-21	87
contig00738	HQ916314.1	97.368	114	3	0	4	117	4008	3895	195	3.70E-46	97
contig00739	HQ916314.1	89.32	103	9	2	16	117	4038	3937	128	3.81E-26	87
contig00741	ON624280.1	87.379	103	11	2	16	117	5982	6083	117	8.25E-23	87
contig00746	KJ620979.1	90	110	11	0	8	117	2835	2726	143	1.36E-30	94
contig00747	ON624284.1	91.892	111	9	0	7	117	3685	3575	156	1.75E-34	95
contig00749	HQ916314.1	89.565	115	10	2	4	117	4044	3931	145	3.78E-31	97
contig00752	HQ916314.1	98.198	111	2	0	1	111	3887	3997	195	3.70E-46	95
contig00753	ON624280.1	90.123	81	8	0	16	96	5982	6062	106	1.79E-19	69
contig00755	HM756260.1	90.123	81	8	0	13	93	2784	2704	106	1.79E-19	69
contig00758	ON624280.1	86.408	103	12	2	16	117	5982	6083	111	3.84E-21	87
contig00759	HQ916314.1	97.297	111	3	0	1	111	3895	4005	189	1.72E-44	95
contig00760	ON624280.1	84.906	106	14	2	13	117	6062	5958	106	1.79E-19	90
contig00762	ON624280.1	86.275	102	14	0	16	117	5982	6083	111	3.84E-21	87
contig00763	ON624280.1	84.906	106	14	2	13	117	6062	5958	106	1.79E-19	90
contig00764	ON624284.1	90.991	111	10	0	1	111	3575	3685	150	8.13E-33	95
contig00765	ON682293.1	81.818	110	20	0	1	110	3304	3413	93.5	1.39E-15	94
contig00770	ON624280.1	84.211	114	16	2	5	117	6070	5958	110	1.38E-20	97
contig00772	ON682293.1	82.727	110	19	0	8	117	3413	3304	99	2.99E-17	94
contig00774	ON624284.1	91.071	112	10	0	6	117	3686	3575	152	2.26E-33	96
contig00776	HM756260.1	90.123	81	8	0	30	110	2704	2784	106	1.79E-19	69
contig00777	HQ916314.1	97.368	114	3	0	1	114	3887	4000	195	3.70E-46	97
contig00781	ON624280.1	87.255	102	13	0	1	102	6083	5982	117	8.25E-23	87
contig00782	HQ916314.1	98.246	114	2	0	4	117	4000	3887	200	7.96E-48	97
contig00787	KJ620979.1	90.909	110	10	0	8	117	2835	2726	148	2.92E-32	94
contig00789	ON624280.1	86.111	108	13	2	1	107	6088	5982	115	2.93E-22	92
contig00791	HM756260.1	90.123	81	8	0	30	110	2704	2784	106	1.76E-19	70
contig00793	ON682272.1	95.413	109	5	0	3	111	3814	3922	174	4.77E-40	94
contig00794	ON624284.1	91.892	111	9	0	1	111	3576	3686	156	1.73E-34	96
contig00796	LC047797.1	93.043	115	8	0	2	116	3548	3662	169	2.22E-38	99
contig00797	ON624280.1	87.037	108	12	2	1	107	6088	5982	121	6.30E-24	92
contig00799	MW373713.1	92.241	116	9	0	1	116	3130	3245	165	2.87E-37	100
contig00800	KJ620979.1	90.517	116	11	0	1	116	2841	2726	154	6.21E-34	100

contig00802	ON624280.1	87.963	108	11	2	1	107	6088	5982	126	1.35E-25	92
contig00806	ON682272.1	96.396	111	4	0	6	116	3817	3927	183	7.92E-43	96
contig00809	ON682272.1	96.226	106	4	0	11	116	3922	3817	174	4.77E-40	91
contig00813	MW373713.1	90.435	115	11	0	2	116	133	19	152	2.23E-33	99
contig00814	ON624280.1	86.916	107	14	0	1	107	6088	5982	121	6.30E-24	92
contig00815	ON624280.1	84.545	110	15	2	2	110	5954	6062	108	4.91E-20	94
contig00817	ON682272.1	95.614	114	5	0	3	116	3814	3927	183	7.92E-43	98
contig00818	KJ620979.1	89.655	116	12	0	1	116	2726	2841	148	2.89E-32	100
contig00820	ON682293.1	83.178	107	18	0	8	114	3413	3307	99	2.95E-17	92
contig00821	MW373713.1	91.304	115	10	0	2	116	133	19	158	4.80E-35	99
contig00823	MW373713.1	90.435	115	11	0	2	116	133	19	152	2.23E-33	99
contig00827	MW373713.1	93.204	103	6	1	14	116	2459	2358	150	8.04E-33	89
contig00828	LC047797.1	92.727	110	8	0	2	111	3548	3657	159	1.34E-35	95
contig00829	ON624280.1	84.545	110	15	2	2	110	5954	6062	108	4.91E-20	94
contig00830	LC047797.1	93.636	110	7	0	2	111	3548	3657	165	2.87E-37	95
contig00834	ON682272.1	96.226	106	4	0	6	111	3817	3922	174	4.77E-40	91
contig00835	ON682293.1	82.243	107	19	0	8	114	3413	3307	93.5	1.37E-15	92
contig00837	LC047797.1	92.174	115	9	0	2	116	3548	3662	163	1.03E-36	99
contig00839	MW373713.1	89.565	115	12	0	2	116	133	19	147	1.04E-31	99
contig00842	HQ916314.1	95.876	97	4	0	19	115	4012	3916	158	4.75E-35	84
contig00843	OQ198051.1	90.351	114	11	0	2	115	3839	3952	150	7.94E-33	99
contig00845	MK378521.1	90.625	64	6	0	5	68	4590	4527	86.1	2.27E-13	56
contig00846	HQ916314.1	94.845	97	5	0	19	115	4012	3916	152	2.21E-33	84
contig00847	MW373713.1	88.393	112	13	0	4	115	3289	3178	135	2.22E-28	97
contig00848	MW373713.1	90.909	110	10	0	6	115	3282	3173	148	2.86E-32	96
contig00852	MW373713.1	90.909	110	10	0	6	115	3282	3173	148	2.86E-32	96
contig00854	MK378521.1	90.625	64	6	0	5	68	4590	4527	86.1	2.27E-13	56
contig00856	MK378521.1	89.855	69	6	1	1	68	4595	4527	87.9	6.31E-14	59
contig00859	MW373713.1	91.892	111	9	0	1	111	3276	3166	156	1.71E-34	97
contig00860	MW373713.1	89.524	105	11	0	1	105	3178	3282	134	8.00E-28	91
contig00863	HQ916314.1	94.565	92	5	0	1	92	3921	4012	143	1.33E-30	80
contig00864	MW373713.1	91.304	115	10	0	1	115	3269	3155	158	4.75E-35	100
contig00868	MW373713.1	92.174	115	9	0	1	115	2468	2354	163	1.02E-36	100

contig00872	ON682293.1	82.301	113	20	0	2	114	3413	3301	99	2.92E-17	98
contig00873	HQ916314.1	98.261	115	2	0	1	115	3997	3883	202	2.16E-48	100
contig00875	MW373713.1	89.286	112	12	0	4	115	3289	3178	141	4.78E-30	97
contig00877	MW373713.1	90.476	105	10	0	1	105	3178	3282	139	1.72E-29	91
contig00881	MW373713.1	92.035	113	9	0	1	113	3276	3164	159	1.32E-35	98
contig00882	MW373713.1	91.589	107	9	0	5	111	3272	3166	148	2.86E-32	93
contig00885	OQ198051.1	90.991	111	10	0	2	112	3839	3949	150	7.94E-33	97
contig00888	HQ916314.1	94.565	92	5	0	1	92	3921	4012	143	1.33E-30	80
contig00890	OQ198051.1	89.474	114	12	0	1	114	3952	3839	145	3.69E-31	99
contig00892	ON682293.1	81.416	113	21	0	2	114	3413	3301	93.5	1.36E-15	98
contig00893	MW373713.1	91.743	109	9	0	5	113	3272	3164	152	2.21E-33	95
contig00895	MW373713.1	92.523	107	7	1	1	107	2354	2459	152	2.21E-33	93
contig00896	HQ916314.1	95.652	92	4	0	24	115	4012	3921	148	2.86E-32	80
contig00897	OQ198051.1	90.09	111	11	0	4	114	3949	3839	145	3.69E-31	97
contig00899	MW373713.1	90	110	11	0	6	115	3282	3173	143	1.33E-30	96
contig00900	HQ916314.1	95.652	92	4	0	1	92	3921	4012	148	2.86E-32	80
contig00901	MW373713.1	90	110	11	0	6	115	3282	3173	143	1.33E-30	96
contig00903	MW373713.1	91.304	115	10	0	1	115	2354	2468	158	4.75E-35	100
contig00904	MW373713.1	93.458	107	6	1	1	107	2354	2459	158	4.75E-35	93
contig00908	HQ916314.1	88.696	115	11	2	1	114	4050	3937	139	1.70E-29	100
contig00911	MZ819164.1	91.071	56	5	0	10	65	4547	4492	76.8	1.35E-10	49
contig00917	MW373713.1	91.228	114	10	0	1	114	3272	3159	156	1.69E-34	100
contig00920	HM756260.1	90.123	81	8	0	19	99	2704	2784	106	1.72E-19	71
contig00922	ON624280.1	90.123	81	8	0	19	99	5982	6062	106	1.72E-19	71
contig00923	HQ916314.1	90.435	115	9	2	1	114	4038	3925	150	7.85E-33	100
contig00924	ON624280.1	90.123	81	8	0	19	99	5982	6062	106	1.72E-19	71
contig00925	ON624280.1	88.764	89	10	0	19	107	5982	6070	110	1.33E-20	78
contig00927	MZ819164.1	91.071	56	5	0	10	65	4547	4492	76.8	1.35E-10	49
contig00929	HQ916314.1	88.696	115	11	2	1	114	4053	3940	139	1.70E-29	100
contig00934	MW373714.1	95.614	114	5	0	1	114	2576	2463	183	7.73E-43	100
contig00936	ON624280.1	90.123	81	8	0	19	99	5982	6062	106	1.72E-19	71
contig00938	HQ916314.1	87.826	115	12	2	1	114	3937	4050	134	7.90E-28	100
contig00941	MW373714.1	95.495	111	5	0	4	114	2573	2463	178	3.60E-41	97

contig00943	ON624277.1	90.351	114	11	0	1	114	2832	2719	150	7.85E-33	100
contig00944	HQ916314.1	88.679	106	10	2	1	105	3940	4044	128	3.68E-26	92
contig00949	JQ696855.1	83.168	101	17	0	1	101	537	437	93.5	1.34E-15	89
contig00952	MW373714.1	94.595	111	6	0	4	114	2573	2463	172	1.67E-39	97
contig00953	HQ916314.1	94.505	91	5	0	1	91	3922	4012	141	4.72E-30	80
contig00954	ON624281.1	87.097	93	12	0	2	94	1067	1159	106	1.72E-19	82
contig00955	MN231240.1	86.087	115	8	4	4	114	183	73	117	7.96E-23	97
contig00957	MW373714.1	94.737	114	6	0	1	114	2576	2463	178	3.60E-41	100
contig00959	OQ198051.1	90.09	111	11	0	4	114	3955	3845	145	3.65E-31	97
contig00963	ON624284.1	90	110	11	0	5	114	3699	3590	143	1.31E-30	96
contig00964	MK378521.1	90.769	65	6	0	50	114	4527	4591	87.9	6.24E-14	57
contig00965	ON682293.1	82.883	111	19	0	4	114	3417	3307	100	8.01E-18	97
contig00968	HQ916314.1	95.604	91	4	0	1	91	3922	4012	147	1.01E-31	80
contig00969	ON624280.1	90.123	81	8	0	19	99	5982	6062	106	1.72E-19	71
contig00970	ON624280.1	87.619	105	13	0	1	105	6086	5982	122	1.71E-24	92
contig00971	ON624280.1	88.764	89	10	0	19	107	5982	6070	110	1.33E-20	78
contig00972	MK378521.1	89.855	69	6	1	47	114	4527	4595	87.9	6.24E-14	60
contig00974	ON682293.1	81.982	111	20	0	4	114	3417	3307	95.3	3.73E-16	97
contig00977	HQ916314.1	87.826	115	12	2	1	114	3940	4053	134	7.90E-28	100
contig00979	MN231240.1	86.441	118	8	4	1	114	70	183	122	1.71E-24	100
contig00981	HQ916314.1	88.991	109	10	2	1	108	3937	4044	134	7.90E-28	95
contig00983	ON624280.1	88.764	89	10	0	8	96	6070	5982	110	1.33E-20	78
contig00986	OQ198051.1	90.351	114	11	0	1	114	3842	3955	150	7.85E-33	100
contig00988	HQ916314.1	96.396	111	4	0	4	114	4012	3902	183	7.73E-43	97
contig00990	OQ198051.1	90.09	111	11	0	1	111	3958	3848	145	3.61E-31	98
contig00994	OQ198051.1	90.09	111	11	0	3	113	3956	3846	145	3.61E-31	98
contig00998	OQ198051.1	90.265	113	11	0	1	113	3846	3958	148	2.79E-32	100
contig01001	ON624280.1	87.156	109	14	0	5	113	5982	6090	124	4.70E-25	96
contig01005	ON682293.1	82.883	111	19	0	1	111	3308	3418	100	7.92E-18	98
contig01006	ON624280.1	87.85	107	13	0	1	107	6088	5982	126	1.31E-25	95
contig01008	ON682293.1	82.883	111	19	0	3	113	3307	3417	100	7.92E-18	98
contig01009	HQ916314.1	90.179	112	9	2	3	113	4038	3928	145	3.61E-31	98
contig01012	HM756260.1	90.123	81	8	0	2	82	2784	2704	106	1.70E-19	72

contig01014	OQ198051.1	90.265	113	11	0	1	113	3836	3948	148	2.79E-32	100
contig01019	ON682293.1	82.883	111	19	0	1	111	3420	3310	100	7.92E-18	98
contig01021	ON624280.1	84.545	110	15	2	2	110	6062	5954	108	4.73E-20	96
contig01022	ON624280.1	94.69	113	6	0	1	113	6120	6232	176	1.28E-40	100
contig01025	ON624280.1	88.073	109	13	0	1	109	6090	5982	130	1.01E-26	96
contig01026	HQ916314.1	95.699	93	4	0	21	113	4012	3920	150	7.75E-33	82
contig01034	ON624284.1	89.908	109	11	0	5	113	3699	3591	141	4.66E-30	96
contig01039	ON682293.1	82.569	109	19	0	3	111	3310	3418	97.1	1.02E-16	96
contig01040	ON624277.1	90.909	110	10	0	1	110	2683	2792	148	2.79E-32	97
contig01044	ON624280.1	88.288	111	13	0	1	111	6092	5982	134	7.80E-28	98
contig01048	HQ916314.1	90.351	114	9	2	1	113	4040	3928	148	2.79E-32	100
contig01051	HQ916314.1	89.474	114	10	2	1	113	4034	3922	143	1.30E-30	100
contig01057	ON682293.1	83.186	113	19	0	1	113	3308	3420	104	6.12E-19	100
contig01059	ON682293.1	81.982	111	20	0	1	111	3417	3307	95.3	3.68E-16	98
contig01061	HQ916314.1	94.624	93	5	0	1	93	3920	4012	145	3.61E-31	82
contig01063	ON624280.1	88.182	110	11	2	5	113	5982	6090	130	1.01E-26	96
contig01065	HQ916314.1	90.351	114	9	2	1	113	4034	3922	148	2.79E-32	100
contig01066	HQ916314.1	98.095	105	2	0	1	105	3893	3997	183	7.64E-43	93
contig01071	MK378521.1	90.625	64	6	0	50	113	4527	4590	86.1	2.22E-13	57
contig01074	MN150125.1	90.654	107	10	0	2	108	3425	3319	143	1.28E-30	96
contig01075	OQ198051.1	90.179	112	11	0	1	112	3845	3956	147	9.90E-32	100
contig01076	MW373713.1	92.793	111	8	0	2	112	3245	3135	161	3.54E-36	99
contig01078	HQ916314.1	97.321	112	3	0	1	112	4004	3893	191	4.51E-45	100
contig01079	MK378521.1	85.057	87	12	1	2	88	4560	4645	87.9	6.09E-14	78
contig01081	MW373713.1	92.793	111	8	0	1	111	3246	3136	161	3.54E-36	99
contig01082	MN231240.1	86.207	87	4	5	3	85	101	183	87.9	6.09E-14	74
contig01083	HQ916314.1	98.148	108	2	0	1	108	3893	4000	189	1.62E-44	96
contig01085	JQ696855.1	83.333	102	17	0	1	102	538	437	95.3	3.64E-16	91
contig01086	MZ603733.1	92.793	111	8	0	2	112	375	265	161	3.54E-36	99
contig01088	ON682293.1	83.036	112	19	0	1	112	3418	3307	102	2.17E-18	100
contig01090	MN231240.1	86.207	87	4	5	28	110	183	101	87.9	6.09E-14	74
contig01091	LC047797.1	91.892	111	9	0	2	112	3520	3630	156	1.65E-34	99
contig01093	MN150125.1	90.654	107	10	0	2	108	3425	3319	143	1.28E-30	96

contig01095	MN231240.1	86.486	111	7	4	6	112	183	77	115	2.79E-22	96
contig01096	MW373713.1	84.545	110	17	0	2	111	1480	1371	110	1.30E-20	98
contig01099	MK378521.1	85.057	87	12	1	25	111	4645	4560	87.9	6.09E-14	78
contig01100	HQ916314.1	95.699	93	4	0	1	93	3920	4012	150	7.65E-33	83
contig01101	LC047797.1	91.892	111	9	0	2	112	3629	3519	156	1.65E-34	99
contig01102	MN150125.1	90.741	108	10	0	5	112	3319	3426	145	3.56E-31	96
contig01103	MW373713.1	84.821	112	17	0	1	112	1481	1370	113	1.00E-21	100
contig01105	ON682298.1	90.991	111	10	0	1	111	2683	2573	150	7.65E-33	99
contig01111	MN231240.1	84.348	115	10	4	2	112	183	73	106	1.68E-19	99
contig01112	HQ916314.1	96.429	112	4	0	1	112	3893	4004	185	2.10E-43	100
contig01113	KJ620979.1	90	110	11	0	2	111	2841	2732	143	1.28E-30	98
contig01114	MW373713.1	84.685	111	17	0	1	111	1481	1371	111	3.61E-21	99
contig01116	MW373713.1	92.857	112	8	0	1	112	3246	3135	163	9.83E-37	100
contig01118	HQ916314.1	90	110	9	2	1	109	3930	4038	141	4.61E-30	97
contig01120	HQ916314.1	90.265	113	9	2	1	112	4035	3924	147	9.90E-32	100
contig01126	MK378521.1	90.769	65	6	0	48	112	4527	4591	87.9	6.09E-14	58
contig01131	ON682298.1	90.991	111	10	0	2	112	2682	2572	150	7.65E-33	99
contig01137	ON682298.1	90.909	110	10	0	2	111	2682	2573	148	2.75E-32	98
contig01142	KJ620979.1	90.09	111	11	0	2	112	2732	2842	145	3.56E-31	99
contig01143	HQ916314.1	96.429	112	4	0	1	112	4012	3901	185	2.10E-43	100
contig01144	ON682298.1	91.071	112	10	0	1	112	2683	2572	152	2.13E-33	100
contig01146	ON682293.1	82.143	112	20	0	1	112	3418	3307	97.1	1.01E-16	100
contig01148	MK378521.1	85.227	88	12	1	1	88	4559	4645	89.8	1.69E-14	79
contig01150	ON682293.1	82.727	110	19	0	1	110	3304	3413	99	2.81E-17	98
contig01151	KJ620979.1	90.909	110	10	0	2	111	2835	2726	148	2.75E-32	98
contig01153	KJ620979.1	90	110	11	0	2	111	2835	2726	143	1.28E-30	98
contig01154	MW373713.1	90.566	106	10	0	7	112	3282	3177	141	4.61E-30	95
contig01157	MK378521.1	85.227	88	12	1	25	112	4645	4559	89.8	1.69E-14	79
contig01160	LC047797.1	91.818	110	9	0	2	111	3520	3629	154	5.92E-34	98
contig01161	MN231240.1	86.207	87	4	5	28	110	183	101	87.9	6.09E-14	74
contig01164	MN231240.1	86.486	111	7	4	2	108	77	183	115	2.79E-22	96
contig01165	HQ916314.1	90.179	112	9	2	1	111	3924	4034	145	3.56E-31	99
contig01167	HQ916314.1	90.09	111	9	2	2	111	4034	3925	143	1.28E-30	98

contig01170	ON624284.1	91.071	112	10	0	1	112	2778	2667	152	2.13E-33	100
contig01173	HQ916314.1	90	110	9	2	4	112	4038	3930	141	4.61E-30	97
contig01174	HQ916314.1	90.179	112	9	2	1	111	4035	3925	145	3.56E-31	99
contig01175	LC047797.1	91.964	112	9	0	1	112	3519	3630	158	4.57E-35	100
contig01176	ON624284.1	91.964	112	9	0	1	112	3686	3575	158	4.57E-35	100
contig01178	ON624284.1	90	110	11	0	1	110	3590	3699	143	1.28E-30	98
contig01180	MW373713.1	92.727	110	8	0	2	111	3245	3136	159	1.27E-35	98
contig01182	MW373713.1	84.685	111	17	0	2	112	1480	1370	111	3.61E-21	99
contig01186	KJ620979.1	90.09	111	11	0	2	112	2841	2731	145	3.56E-31	99
contig01188	MN150125.1	90.741	108	10	0	1	108	3426	3319	145	3.56E-31	96
contig01189	HQ916314.1	94.624	93	5	0	1	93	3920	4012	145	3.56E-31	83
contig01190	HQ916314.1	97.321	112	3	0	1	112	4005	3894	191	4.51E-45	100
contig01192	MN231240.1	86.207	87	4	5	28	110	183	101	87.9	6.09E-14	74
contig01193	ON682293.1	81.818	110	20	0	3	112	3413	3304	93.5	1.31E-15	98
contig01197	MN231240.1	86.207	116	8	4	1	112	73	184	119	2.16E-23	100
contig01200	KJ620979.1	90.179	112	11	0	1	112	2842	2731	147	9.90E-32	100
